# Cell jamming in a collagen-based interface assay is tuned by collagen density and proteolysis

**DOI:** 10.1242/jcs.260207

**Published:** 2023-12-15

**Authors:** Lianne Beunk, Nan Wen, Sjoerd van Helvert, Bram Bekker, Lars Ran, Ross Kang, Tom Paulat, Simon Syga, Andreas Deutsch, Peter Friedl, Katarina Wolf

**Affiliations:** ^1^ Department of Medical BioSciences, Radboud University Medical Center, Nijmegen, GA 6525, The Netherlands; ^2^ Department of Mathematics, Faculty of Natural Science, Mathematics and Informatics, Radboud University, 6500 GL Nijmegen, The Netherlands; ^3^ Department of Innovative Computing, Centre for Information Services and High Performance Computing, Technical University Dresden, 01062 Dresden, Germany; ^4^ David H. Koch Center for Applied Genitourinary Cancers, The University of Texas MD Anderson Cancer Center, Houston, TX 77030, USA

**Keywords:** Collagen–collagen cleft model, Tumor spheroid, Cell migration, Cell jamming, Linear confinement, ECM-directed proteolysis

## Abstract

Tumor cell invasion into heterogenous interstitial tissues consisting of network-, channel- or rift-like architectures involves both matrix metalloproteinase (MMP)-mediated tissue remodeling and cell shape adaptation to tissue geometry. Three-dimensional (3D) models composed of either porous or linearly aligned architectures have added to the understanding of how physical spacing principles affect migration efficacy; however, the relative contribution of each architecture to decision making in the presence of varying MMP availability is not known. Here, we developed an interface assay containing a cleft between two high-density collagen lattices, and we used this assay to probe tumor cell invasion efficacy, invasion mode and MMP dependence in concert. *In silico* modeling predicted facilitated cell migration into confining clefts independently of MMP activity, whereas migration into dense porous matrix was predicted to require matrix degradation. This prediction was verified experimentally, where inhibition of collagen degradation was found to strongly compromise migration into 3D collagen in a density-dependent manner, but interface-guided migration remained effective, occurring by cell jamming. The 3D interface assay reported here may serve as a suitable model to better understand the impact of *in vivo*-relevant interstitial tissue topologies on tumor invasion patterning and responses to molecular interventions.

## Teaser

**Summary:** We report an interface assay that mimics the guiding inner surfaces of 3D interstitial tissues, providing a model for *in vivo*-relevant studies of tumor cell invasion and responses to molecular intervention.

## INTRODUCTION

Interstitial tissues connect and support organ-specific tissues in the body and are rich in extracellular matrix (ECM) components, with collagen forming the predominant supportive architectures. Due to the variety in their composition and function, interstitial compartments are highly heterogenous in density, topology and stiffness (Charras and Sahai, 2014; van Helvert et al., 2018). In the interstitium of the upper skin, for example, collagen fibers form a range of structures that includes one-dimensional (1D) line-like single-fiber structures as well as three-dimensional (3D) random network-like topologies, and harbor components such as blood vessels, nerves and glands. In contrast, the lower dermis consists of densely packed collagen in stiffer cable-like structures, and neighboring tissues, such as muscular layers or perineural tracks, can feature long linear structures (Weigelin et al., 2016). These tissue architectures form topologies that contain narrow cleft-like interfaces and tunnels of linear geometry that are often hundreds or thousands of micrometers in length and are filled with interstitial fluid, glycosaminoglycans, small vessels, or low-density networks of fibrillar collagen or other ECM components. Usually, these interstitial tissue spaces range between 0.5 µm and 30 µm in diameter and have cross-sections of 0.25–900 µm^2^ (Benias et al., 2018; Weigelin et al., 2012). To mimic aspects of the versatile architectures present in interstitial tissues, reconstituted or cell-derived collagen-based models have been developed, ranging from 3D fibrillar networks of random orientation, varying porosity and fibril thickness to lineary aligned fibrillar structures, tunnel-like tracks in hydrogels and under-collagen assays (Beunk et al., 2022; Carey et al., 2015; Ilina et al., 2020; Raub et al., 2007; Ray et al., 2018; Shinsato et al., 2020; Weigelin et al., 2021; Zanotelli et al., 2019). However, a model combining an interface with thicker collagen layers, mimicking neighboring collagen bundles and networks, is lacking.

During metastatic invasion of cancer (for example, invasion of melanoma into the dermis) cells penetrate complex ECM architectures, which can function as both inhibitors and promotors of cell locomotion by providing barrier-like or guiding cues, respectively (Charras and Sahai, 2014). Cells negotiate spaces in dense architectures by either adapting their shape, including that of the spacious and stiff nucleus, to the pre-existing space, or by tumor-related proteolytic matrix metalloproteinase (MMP)-derived ECM remodeling to overcome resistance imposed by high-density matrix (Fröhlich, 2010; Haeger et al., 2014; Krause and Wolf, 2015; McGregor et al., 2016; Wolf et al., 2013). As a result of the latter process, barrier-free tracks are generated that laterally confine and guide cells, and these tracks gradually widen and become filled by follower cells, leading to the phenomenon of cell jamming (Gaggioli et al., 2007; Ilina et al., 2020; Wolf et al., 2007). This process results in the formation of collective strands of cells surrounded by dense ECM that, depending on the origin of the cells, might form functional cell–cell junctions, such as those mediated by ALCAM or other candidate adhesion systems (Haeger et al., 2014; Ilina et al., 2020; Venhuizen et al., 2019). On the other hand, preformed tissue-intrinsic track-like tunnels and clefts *in vivo* and *in vitro* can provide linear cues to guide migrating cells, enhancing the efficacy of cell motility (Beunk et al., 2022; Carey et al., 2015; Ilina et al., 2020; Weigelin et al., 2012; Zhu et al., 2015). Depending on the density and stiffness, and thus compressibility, of the surrounding tissues, spaces can widen to some extent to harbor migrating cells that then form jammed collectives even in the absence of proteolytic matrix remodeling (Ilina et al., 2011; Tarle et al., 2017). Whereas cell patterning into tissue compartments with porous or linearly guiding architectures has been studied previously (Ilina et al, 2020; Wolf et al., 2013), how tumor cells choose between ECM geometries for 3D invasion depending on the presence or absence of guidance cues and the ability to degrade ECM remains unclear. Here, we developed a high-density collagen-based *in vitro* assay that contains a low-density guiding cleft, and we tuned the assay by reducing the collagen density and inhibiting MMPs, which allowed us disentangle the relative impact of ECM-derived barriers against interface guidance. The assay appeared suitable to demonstrate (1) that MV3 melanoma cells choose between different ECM geometries in a manner that is dependent on ECM density and MMP availability, and (2) that pronounced linear cues within high-density ECM enable non-proteolytic cell jamming in an otherwise migration-prohibitive environment.

## RESULTS

### Establishing a collagen–collagen interface migration model

**Fig. 1.**
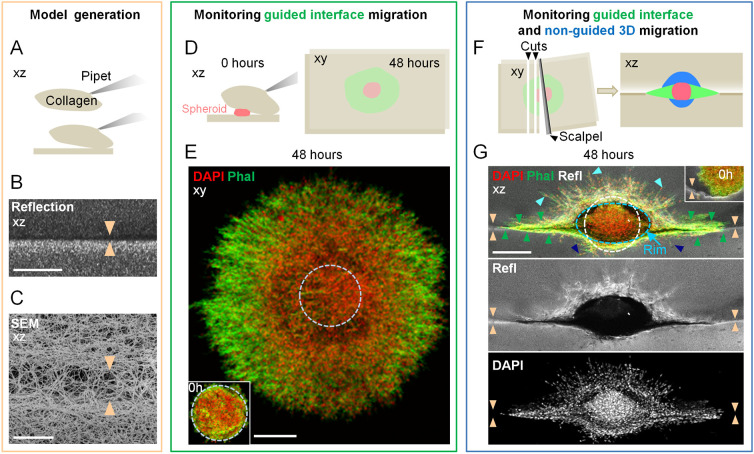
**Emigration of MV3 melanoma cells from spheroids into the 3D interface model.** The collagen-collagen interface model (A–C), with embedded spheroids after 0 h and 48 h (D–G). (A) Cartoon depicting the principle of interface model generation. (B,C) Visualization of the collagen–collagen interface (indicated by arrowheads in the *xz* side views) consisting of low collagen density between two high-density (6 mg/ml) collagen lattices, as monitored by confocal reflection microscopy (B) or SEM (C). (D) Cartoons depicting the principle of (left) spheroid embedding and (right) interface emigration (shown in green in the *xy* top view). (E) Imaging of cell emigration into the interface at 48 h, shown as a top view (*xy*). Dashed circle indicates the outline of the original spheroid core directly after collagen polymerization (see inset). Phal, phalloidin. (F) Schematics depicting (left) the manual cross-section method and (right) a resulting side view of migration patterns (interface migration, green; migration into porous 3D collagen network, blue; spheroid, red). (G) Visualization of cell emigration from a spheroid into the interface (green arrowheads), and into the lower (dark blue arrowheads) and upper (light blue arrowheads) 3D collagen lattices. Orange arrowheads indicate the interface. The dashed circle depicts the average initial spheroid size (see inset for spheroid size at 0 h culture between collagen lattices); the flattened spheroid within a hollow core after 48 h is indicated by the fitted blue dashed ellipse, defined as the spheroid rim. Refl, reflection. Scale bars: 250 µm in B,E and G; 5 µm in C.

To obtain a collagen-based assay that consisted of both a porous component and a guiding component, we polymerized a high-density (6 mg/ml) 3D collagen lattice and subsequently reconstituted a second collagen gel on top (Fig. 1A). The bottom and top lattices formed two interfaces with a cleft between them that was initially ∼10 µm in height and consisted of bridging collagen fibrils at low density, which we collectively refer to as the ‘interface’ (Fig. 1B,C; Fig. S1A,B). The densities of the interface boundaries, which guided and confined cells in the assay, were asymmetric, with higher density beneath the interface. The collagen-compacted upper layer of the bottom gel, when compared to the cleft, had a 3–4-fold increased collagen reflection signal, and the remaining layers of the bottom gel, as well as the top gel, had an ∼2-fold increased reflection signal (Fig. 1B; Fig. S1A,C). The mechanism for this asymmetry in density is not known, but we assume that compression by the weight of the top gel, but not effects of water evaporation or gravity, contributed to condensation of the lower collagen interface. Next, MV3 melanoma spheroids were embedded between the two gel layers (Fig. 1D, left), and these spheroids remained intact without detached cells being present directly after gel reconstitution (Fig. 1E,G, insets), confirming that spheroid morphology was largely unaffected by the constitution process. Thus, a collagen model comprising a confining linear interface adjacent to randomly polymerized 3D fibrillar networks for spheroid culture was developed.

The interface assay enabled us to investigate the effect of ECM geometry on cell patterning over a 48 h period (Fig. 1D,F). Melanoma emigration into the interface occurred over an area of ∼0.75 mm^2^, which was 5–10 times larger than the initial spheroid size (Fig. 1E; Fig. S2A,B). To dissect migration patterns in the different layers of the collagen assay, we established a methodology to cross-section the lattices and image these at a 90° angle (Fig. 1F; Fig. S3A). Notably, the size of the spheroid remained largely constant over 24–48 h, possibly due to effective cell emigration, which occurred to such a degree that an occasional hollow zone emerged around the spheroid (Fig. 1F,G, see ellipse in blue; Fig. S2A,C).

By imaging from the side, we observed efficient sheet-like cell positioning along the interface, as well as emigration into the 3D matrix both above and below the spheroid center in the form of collective strand-like patterns (Fig. 1G). The interface-guided migration patterns consisted of ∼2000 cells that migrated over distances of up to ∼400 µm (Fig. S2D–F). Cell migration into the 3D collagen was more frequent in the top gel, as compared to that in the lower collagen compartment, whereas a similar non-linear, non-logarithmic decrease in the distribution of cells over a distance of ∼175 µm into the lattice from the rim of the spheroid core occurred for both layers (Fig. S3A–C). Presumably, the presence of the high-density collagen layer reduced the number of cells entering the bottom matrix, but once access was gained, similar migration kinetics occurred in both lattices (Fig. S3C, bottom). After cell counting and application of an imaging-related correction factor (Fig. S3A; see Image analysis section in the Materials and Methods), we found that 350 and 150 cells migrated into the top and bottom layer, respectively, and thus on average a total of 500 cells migrated into the 3D collagen (Fig. S3D). Taking all cells that left the tumor spheroid together, the interface harbored around four times as many cells as the 3D collagen lattices (2000 cells versus 500 cells; Fig. S2E, Fig. S3D), and cells in the interface migrated ∼2.5 times further than those in the lattices (400 µm versus 150-175 µm; Fig. S2F; Fig. S3C). Thus, the interface within a high-density matrix environment supported prominent cell migration behavior in terms of both numbers and distance.

### Cell migration along 3D clefts results in a jamming-like phenotype

**Fig. 2.**
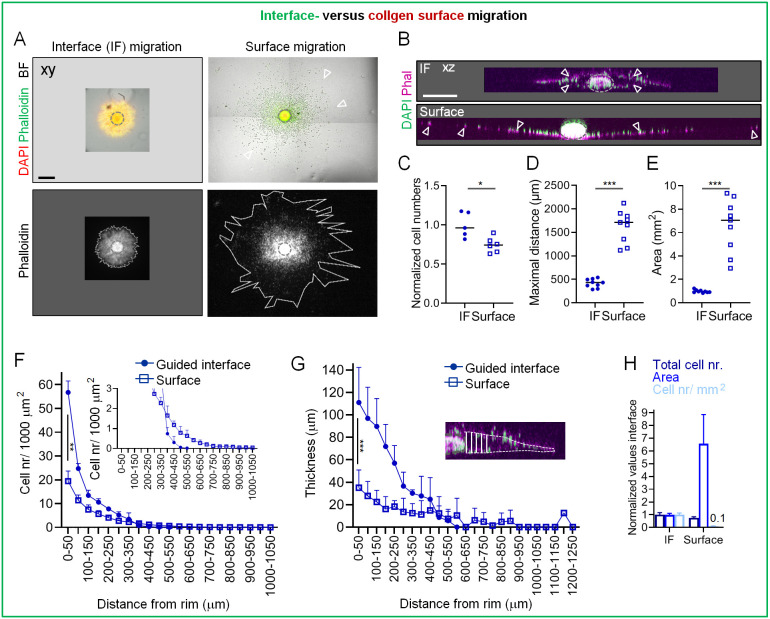
**MV3 cell jamming into the cleft versus migration over a collagen surface.** Invasion from the spheroid in the interface (IF) assay and over the surface of a 3D collagen droplet (both 6 mg/ml collagen) in the presence of DMSO were monitored after 48 h of culture. (A) Top views of interface and collagen surface migration. Gray dashed circles indicate the spheroid rim; white outlines (lower row) delimit cell-populated areas; open arrowheads mark examples of single cells that migrated on the collagen surface. BF, bright-field signal. (B) Projected side views of cells that migrated into the interface (top) or over the collagen surface (bottom). Gray dashed ellipses show spheroid rims, as described for Fig. 1G; arrowheads mark emigrated cells. Phal, phalloidin. (C–E) Quantification of interface- and surface-guided migration parameters outside the spheroid rim. Normalized number of emigrated cells (C), maximum migration distance (D) and area of migration (E) are shown. Solid line, median. Data are pooled from *N*=2–3 experiments, with between one and five measurements per condition per experiment (each data point represents a spheroid). (F) Quantification of cell numbers in relation to the distance from the spheroid rim, measured from binned sections increasing by 50 µm radius around the rim (see Fig. S2D,E). Inset graph shows a zoom-in of the *x*-axis in relation to the distance from the rim. *N*=2–3, with between one and five measurements per condition per experiment. Nr, number. (G) Thickness of the cell invasion zone into the cleft or over the collagen surface at the indicated distances from the spheroid rim. Inset, depiction of measurement method, showing a region of the interface migration image in B. Dashed white line marks the invasion zone in the cleft; solid white lines indicate the thickness of the invasion zone. Data are pooled from *N*=2–3 experiments, with between one and five measurements per condition per experiment. (H) Depiction of normalized total cell numbers and the measured area of interface and surface migration (data are from C and E), and the resulting cell density. Data are pooled from *N*=2–3 experiments, with between one and five measurements per condition per experiment. Data in F–H are presented as mean+s.d. *P<0.05; **P<0.01; ***P<0.001 (two-tailed unpaired Mann–Whitney test in C–E; two-tailed unpaired *t*-test in F and G). Scale bars: 500 µm in A; 250 µm in B. Images and data from this figure are also shown in Fig. S5A–H for comparison with assays using the GM6001 inhibitor.

To address whether the effective migration into the interface was caused by specifics of the 3D cleft – for example, dual contact with the upper and lower interface – we performed control experiments that involved seeding spheroids on top of a high-density collagen gel without an additional collagen layer. In this 2.5D-like setting, cells on the collagen gel surface migrated both in bulk and as single cells in numbers similar to those observed for migration into the cleft, but covered up to 3.5-fold increased distance relative to the migration distance in the cleft, resulting in a 7-fold larger coverage area compared to that observed for interface migration (Fig. 2A,C–F; Fig. S5A,C–F). As a consequence of 3D confinement, we observed a transition to a multilayered sheet organization of ∼3-fold increased thickness and with an overall 10-fold increased cell density, whereas unconfined migration across the collagen surface yielded a monolayer in the majority of cases (Fig. 2B,F–H; Fig. S5B,F–H). Taken together, these observations reveal that the interface in a high-density matrix environment guides migration of large cell numbers into limited space, leading to cell jamming.

### Switch in invasion efficiency in response to ECM density and topology

**Fig. 3.**
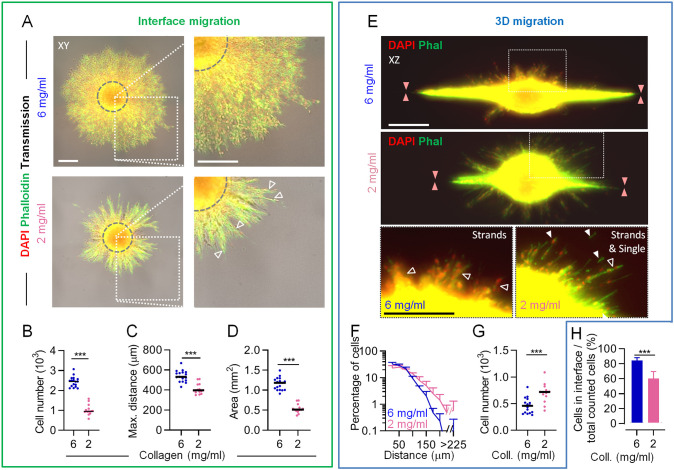
**Altered migration patterning in collagen–collagen interface lattices of different densities.** Invasion from spheroids in the interface assay at the indicated collagen densities and in the presence of DMSO was monitored after 48 h culture. (A) Top view for the monitoring of interface migration. Gray dashed circles indicate size of the depicted spheroid when imaged at 0 h. Rectangles indicate regions shown in expanded views in the right column. Open arrowheads indicate collective strands. (B–D) Quantification of interface-guided migration parameters for assays as described in A. Number of emigrated cells (C), maximum migration distance (D) and area of migration (E) are shown. Solid line, median. Data are pooled from *N*=4 experiments, with between one and five measurements per condition per experiment (each data point represents a spheroid). (E) Side view for the monitoring of 3D invasion. Rectangles indicate regions shown in expanded views in the bottom row. Orange arrowheads, location of the interface; open arrowheads, collective strands; filled arrowheads, single cells. Phal, phalloidin. (F,G) Quantification of (F) distribution and (G) total numbers of cells in porous 3D lattices of the indicated collagen (Coll) concentrations. (H) Proportion of cells in the interface, expressed as a percentage of total numbers of migrated cells (into interface and 3D collagen) per spheroid. Data are pooled from *N*=4 experiments, with between one and five measurements per condition per experiment. (B–D,G) Solid line, median; (F,H) mean+s.d. ***P<0.001 (two-tailed unpaired Mann-Whitney test). Scale bars: 250 µm. Images and data from A, B and D are also shown in Fig. S4A–C for comparison with assays using the GM6001 inhibitor.

To challenge the process of cell jamming by varying the extent of interface formation, we next investigated how reduced collagen density and the resulting interface architecture impacted decision making of invading tumor cells. Decreasing the concentration of collagen to 2 mg/ml resulted in a less prominent cleft, characterized by a mildly reduced height (5–8 µm), with only ∼10% difference in collagen reflection signal intensity between the  cleft and the upper 3D matrix, and only a 1.6-fold increase in collagen reflection signal between the cleft and the rim of the bottom gel (Fig. S1D–F). In consequence, cell invasion along the interface bordered by low-density matrix switched from the sheet-like migration observed in the high-density assay to a collective strand pattern (Fig. 3A). Compared to interface migration in the high-density assay, these strands consisted of 60% fewer cells and covered 60% less area, thus maintaining an unaltered overall cell density, and migrated ∼20% less far (Fig. 3B–D; Fig. S4). In contrast, the 3D collagen compartments of the low-density assay were infiltrated by thin strands and, occasionally, single cells, with up to 1.5-fold increased migration distance and up to 1.5-fold increased cell numbers relative to the more compact and shorter strands observed to infiltrate these compartments in the high-density assay (Fig. 3E–G). This led to a decrease of ∼25% in the relative fraction of cells migrating along the interface in the low-density assay (Fig. 3H), demonstrating that the less defined interface of the low-density ECM assay supports jamming-like migration behavior at a smaller scale.

### *In silico* prediction of cell invasion in response to ECM density, topology and proteolysis

**Fig. 4:**
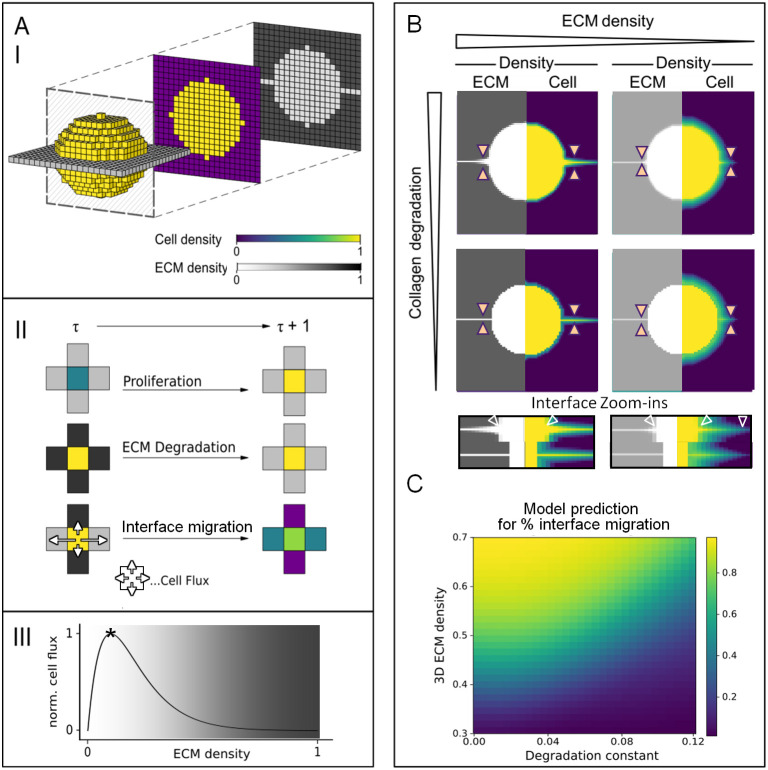
***In silico* cellular automaton-based modeling of tumor cell invasion into a cleft.** (A) Development and (B,C) application of an *in silico* model predicting tumor cell invasion into a low-density cleft. The color scheme depicted in panel I of A applies to all panels in A and B. (A) Panel I: a 3D lattice with each voxel containing scalar values for cell and ECM density ranging from 0 to 1. Initial conditions of a high-density cell spheroid and low-density ECM interface are shown. Panel II: depiction of discrete timestep-dependent (*τ*→*τ*+1) deterministic changes of ECM and cell densities due to proliferation, ECM degradation and interface migration. In the lowest depicted scheme, cells adjust their local movement direction along the axis of low ECM density, here implemented as cell density increase caused by cell flux into the target voxel (see arrows). Panel III: dependency of cell flux on ECM density. The critical ECM density is indicated by an asterisk. (B) Depiction of ECM density (left side of each diagram) and cell density (right side of each diagram) after 100 time steps at varying collagen degradation and ECM density values. Orange arrowheads indicate the location of the cleft. Bottom row shows expanded views of the interface for the four conditions shown above, with arrowheads indicating the impact of collagen degradation on ECM and cell density. (C) Prediction of the percentage of interface-guided migration as related to total migration (scaled from 0 to 1) as a function of ECM density and ECM degradation.

To predict the consequences of ECM degradation for cell migration along the cleft, we applied an *in silico* cellular automaton model in which we simulated cell migration from a spheroid into a linear space between two 3D collagen meshworks (Fig. 4A, panel I). Cell densities changed over time according to a deterministic interaction rule that incorporated proliferation, matrix degradation and ECM guidance (Fig. 4A panels II and III), resulting in patterns of cell density similar to the characteristic invasion patterns we observed *in vitro* (Fig. 4B; Movie 1). The model predicted that with increasing 3D ECM densities and decreasing degradation rates the percentage of cells migrating along the interface relative to all emigrating cells will increase (Fig. 4C). When fitting the model to our observations from individual experiments, we found that the cells would be required to degrade collagen ∼2.5 times faster in the high-density collagen condition than in the low-density collagen condition to achieve the observed invasion patterns, which is in good agreement with previous experimental findings (Infante et al., 2018). Overall, the cellular automaton model allowed us to predict that without collagenolysis, migration along the interface between high-density collagen lattices would continue and be most dominant.

### Guided interface migration enables non-proteolytic migration

**Fig. 5.**
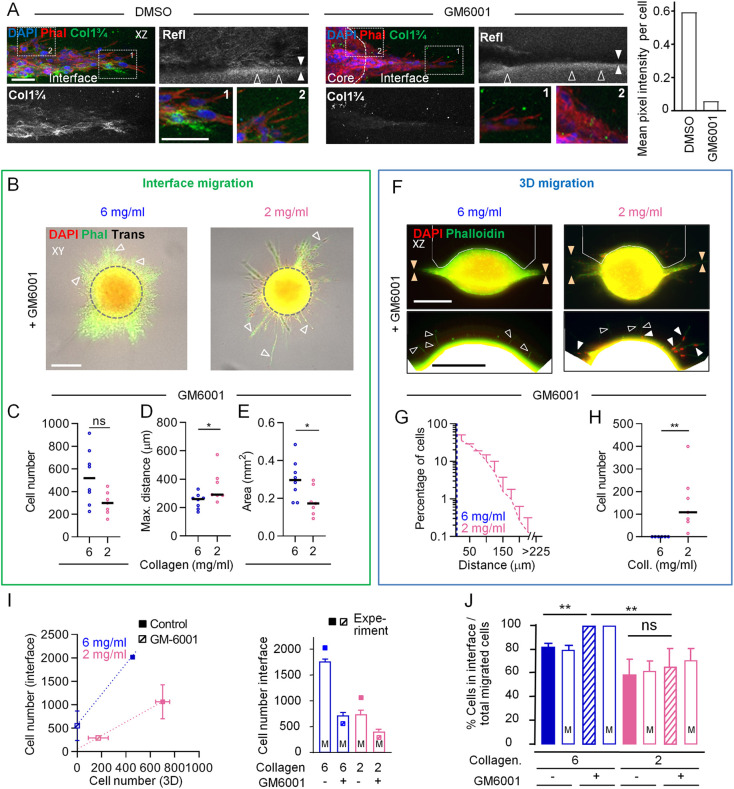
**Differential MMP-dependence of invasion along interfaces or into 3D collagen.** Cell emigration patterns in the absence or presence of MMP inhibitor GM6001 after 48 h culture were monitored and quantified. (A) Side view of cleft migration in high-density collagen in the presence of either 5 µM GM6001 or DMSO as a vehicle control. Where present, the rim of the spheroid core is indicated by a white dashed line. Numbered dotted rectangles mark regions shown in the zoom-in images. Open arrowheads indicate increased reflection signal from the rim of the lower gel; filled arrowheads indicate the interface. The graph shows the mean signal of the collagen 1 three-quarter fragment (Col1 ¾) per cell from the interface (DMSO, 75 nuclei; GM6001, 118 nuclei; *N*=1). Phal, phalloidin; Refl, reflection. (B–H) Visualization and quantification of cell emigration in the presence of 5 µM GM6001. (B) Top views of cell emigration in the indicated conditions. Gray dashed circles indicate the size of the depicted spheroid when imaged at 0 h culture. Arrowheads, strand-like migration. Trans, transmitted light. (C–E) Quantification of interface-guided migration parameters in the presence of GM6001. Number of emigrated cells (C), maximum migration distance (D) and area of migration (E) are shown. (F) Side views of emigration in the indicated conditions. Areas bordered by white lines in the top images are shown in expanded images beneath. Orange arrowheads, cleft; open arrowheads, cytoplasmic extensions; filled arrowheads, invaded cell nuclei. (G,H) Quantification of non-guided migration parameters. Migration distance (G) and number of emigrated cells (H) for the indicated collagen (Coll.) concentrations are shown. Data in C–E, G and H are pooled from *N*=2 experiments, with between one and four measurements per condition per experiment*.* (I,J) Interface and 3D migration in the presence or absence of MMP activity. All bars depicted with ‘M’ were derived from the *in silico* cellular automaton-based model. All other data were derived from experiments depicted in Fig. S4B,E. (I) Left: total numbers of interface-guided cells plotted against numbers of 3D non-guided cells for the indicated conditions. Right: agreement of model prediction (bars) with experimental values (points) of the number of interface-guided cells in the indicated conditions. (J) Emigrated cells in the cleft as a percentage of total emigrated cell numbers per spheroid, comparing experimental values with those predicted by the model. *N* = 2–4 replications for experimental values. Solid lines in C–E and H indicate the median; Data in G,I and J are presented as mean+s.d. *P<0.05; **P<0.01; ns, not significant (two-tailed unpaired Mann–Whitney test in C–E, H; two-tailed unpaired Mann–Whitney test with Holm–Sidak test in J). Scale bars: 50 µm in A; 250 µm in B and F. Images and data from B, C, E, F and H are also shown in Fig. S4 for comparison with control assays using DMSO.

To evaluate the prediction of the model regarding sustained non-proteolytic infiltration of the interface but not the 3D ECM, we applied the broad-spectrum MMP inhibitor GM6001 and monitored invasion. Extensive collagen degradation by MV3 cells invading along the interface was reduced by ∼90% after MMP inhibition (Fig. 5A). Consistent with moderate hindrance imposed by the matrix upon MMP inhibition (Infante et al., 2018; Wolf et al., 2013), during cleft migration between high-density collagen lattices in the presence of GM6001, cell numbers, covered area, migration distance and invasion zone thickness were decreased compared to those in the control condition (Fig. S4A–C; Fig. S5A–E,H–M,P), but the cell density in the cleft itself, as a measure for cell jamming, was unaffected (Fig. S5G,O). This jamming phenomenon, indicated by remaining multilayered migration, was again evident when compared to non-proteolytic migration of monolayered cells across the unconfined collagen surface (Fig. S5I–P). Lastly, at both high and low collagen densities, non-proteolytic cleft migration numbers and areas decreased by 50–75% when compared to those observed for proteolytic migration, and cells migrated similar distances (Fig. 5B–D; Fig. S4A,B). Interestingly, the cell numbers and covered area observed for non-proteolytic migration into the cleft in high-density collagen conditions showed a 1.7-fold increase over those observed in the low-density collagen conditions, indicating a greater efficacy of residual jamming in the high-density conditions (Fig. 5C,E; Fig. S4B,C). Taken together, cell jamming, the phenomenon of cells being confined into limited space surrounded by high-density substrate, depends on high spatial linear confinement over MMP-mediated ECM degradation (Fig. S5H,P).

In contrast to interface patterning, migration into the high-density 3D collagen matrix was abolished in the presence of GM6001 (Fig. 5F–H; Fig. S4D,E), as characterized by the appearance of only cytoplasmic protrusions, and not cell nuclei, originating from tumor core-derived cells in the matrix (Fig. 5F, lower left image). Residual migration into low-density collagen, on the other hand, was maintained in the presence of GM6001, as evidenced by occasional strand-like cell patterns that also included cell nuclei (Fig. 5F, lower right image). In summary, non-proteolytic migration into the 3D high-density matrix was inhibited in accordance with the physical limit of migration (Wolf et al., 2013). In contrast, non-proteolytic migration was enabled by guiding cues present within the high-density matrix and, to a lower degree, in the low-density assay, where the guiding matrix cues were poorly defined (Fig. 5I, left). Hereby, the *in vitro* data confirmed the *in silico* prediction (Fig. 5I, right) that even without MMP activity the interface between high-density tissues enables more migration compared to that occurring between low-density matrices. Consequently, whereas in the low-density assay the percentage of emigrating cells that migrated along the cleft was unchanged upon MMP inhibition, spheroid emigration in the high-density assay shifted completely towards interface-guided migration (Fig. 5J), in accordance with the prediction of our model.

## DISCUSSION

Here, we developed an *in vitro* spheroid invasion assay consisting of two layers of 3D fibrillar collagen that formed a guiding cleft of confined topology but low collagen density. By employing a newly developed cross-sectional approach, cell invasion could be visualized effectively, allowing quantification of the impact of substrate density, topology and MMP-dependent collagenolysis on decision-making for invasion into the different ECM compartments. Our *in vitro* and *in silico* data show that the presence of a low-density cleft provides both guidance and mechanical resistance, thereby inducing cell jamming to (1) support cell motility and (2) enable MMP-independent migration.

### A confining cleft in high-density collagen facilitates migration but leads to cell jamming

Migration in our *in vitro* interface assay occurred predominantly along 5–10 µm-high clefts of low ECM density bordered by high-density matrix, reminiscent of largely linear interfaces and clefts occurring in interstitial tissue *in vivo* (Benias et al., 2018; Weigelin et al., 2012). Our electron microscopically derived imaging further revealed bridging collagen fibrils within the cleft, which we estimated to have a 2–10-fold greater pore size than the surrounding 3D collagen (Fig. 1C). Even though precise pore size measurements are not possible, as such collagen gels shrink during sample processing for electron microscopy imaging and the axial resolution of confocal reflectance microscopy is insufficient to address the pores in collapsed cell-free clefts, cell behavior in the presence of GM6001 indicates that these pores might have an area of 8–20 µm^2^ (Wolf et al., 2013). As a migration-promoting characteristic, the cleft was bordered by a rim of high density, presumably induced by adding weight from a second collagen layer, which is a phenomenon that we consider intrinsic to the generation of our interface collagen model. High density corresponds to increased local ECM stiffness; an increase in collagen concentration from 2 mg/ml to 8 mg/ml has been shown to induce an increase in stiffness from below 100 Pa up to 600 Pa (Haeger et al., 2014; Wolf et al., 2013), a range that has been found previously to positively impact cell migration rates (Ulrich et al., 2009). Furthermore, linear confinement supports and guides cell migration via cellular polarization and induction of alignment of the actin–myosin network and focal adhesions in the direction of the geometrical cue (Mosier et al., 2019; Ray et al., 2017; Tarle et al., 2017), all mechanisms that we propose promote interface migration along the high-density rim. On the other hand, in accordance with previous observations of jammed migration along confined space (Haeger et al., 2014; Ilina et al., 2020), we show here that the confining cleft of our interface model, although promoting migration by the mechanisms described above, ultimately induces cell jamming. Thus, by direct comparison to the monolayer migration emerging on an unconfined surface of a collagen lattice, the role of confinement in the cleft to induce multilayered cell jamming is now emphasized.

### Role of collagenolysis in matrix invasion

Mesenchymal cancer cell patterning within 3D dense matrices, combined with partial breakdown of surrounding matrix, is associated with as much space generation as necessary to accommodate invasive growth (Haeger et al., 2014; Wolf et al., 2007). Matrix degradation and proliferation, for example, are coupled such that collagenolysis creates the space necessary for cell expansion (Hotary et al., 2003). This ‘space generation’ principle also applies to cell migration in very dense matrices, where MMP activity is mandatory for the cell to move forward (Hotary et al., 2003; Sabeh et al., 2004; Wolf et al., 2013), with the amount of matrix degradation depending on matrix density, consistent with a ‘digest-on-demand’ mechanism (Infante et al., 2018). Cancer cells with active MMP systems can thus invade a broad range of tissues, even within restrictive high-density areas. However, in the absence of MMPs, both single and collective cell motility into spaces within dense 3D matrices is delayed or even abrogated (Sabeh et al., 2004; Wolf et al., 2013), and is determined by the deformation limit of the relatively stiff cell nucleus, thereby defining non-guided migration into dense 3D matrix as an MMP-dependent process. By contrast, we observed that migration along low-density clefts between high-density matrices persists in the absence of collagenolysis, enabling us to identify residual non-proteolytic cell jamming as an MMP-independent invasion niche, similar to our previous findings in tissue track models and under-collagen assays (Beunk et al., 2022; Ilina et al., 2020). Taken together, our findings indicate that linearly confined low ECM density within dense ECM supports jamming migration in both the presence and absence of MMP activity, thereby defining ECM guidance as a primary determinant of cell patterning above matrix degradation.

### Implications

To conclude, we characterized distinct cell migration modes in our newly developed interface assay, with the aim of modeling cancer cell migration in *in vivo* tissues with regard to biophysical properties such as varying density and geometry. The interface assay complements collagen-based track models, although an important difference is that the tracks are consistently devoid of solid structures (Beunk et al., 2022; Zanotelli et al., 2019). The fact that the interface contains low-density collagen fibrils adds an important component to better mimic linear spaces of reduced resistance perineural tracks, along or within muscular layers, or between dense interwoven collagen bundles, as well as the spaces forming tissue conduits (Benias et al., 2018; Beunk et al., 2019; Weigelin et al., 2012; and our unpublished observations). Accordingly, the finding that in the absence of collagenolysis cancer cell locomotion along tissue interfaces persists, even though at reduced rates, implies that invasion into cleft-like matrix structures *in vivo* is not sufficiently responsive to protease inhibitor therapy, whereas invasion into densely structured but randomly organized tissue is predicted to be halted. Therefore, this interface assay can be applied together with *in silico* modeling and further developed to dissect the impact of complex tissue spacing *in vivo* on cancer cell invasion patterns in response to therapy, thereby complementing and providing an alternative to animal experiments.

## MATERIALS AND METHODS

### Antibodies and inhibitors

Affinity-purified rabbit anti-collagen type I cleavage site antibody (Col1¾C_short_; Immunoglobe, 0217-025; 1:500) (Wolf et al., 2013), Alexa Fluor 488-conjugated goat anti-rabbit IgG pre-absorbed secondary antibody (Invitrogen, A11034; 1:200), DAPI (Sigma) and Alexa Fluor 568-conjugated phalloidin (Invitrogen) were used. To block collagen degradation, the broad-spectrum MMP inhibitor GM6001 (dissolved in DMSO; Calbiochem) was used at a final concentration of 5 µM.

### Cell culture and spheroid generation

Human wild-type MV3 melanoma cells, kindly provided by G. van Muijen (Department of Pathology, Radboud University Medical Center, Nijmegen; van Muijen et al., 1991), were cultured (humidified atmosphere, 10% CO_2_, 37°C) in Dulbecco's modified Eagle's serum medium (DMEM, Invitrogen) supplemented with 10% fetal calf serum (Sigma), penicillin (100 U/ml, PAA), streptomycin (100 µg/ml, Invitrogen), L-glutamine (2 mM, Lonza) and sodium pyruvate (1 mM, Gibco). Identity of the MV3 cells was verified by short tandem repeat (STR) DNA profiling (IDEXX BioResearch), and no mammalian interspecies contamination was detected. Lack of contamination with mycoplasma was routinely verified using the MycoAlert Mycoplasma Detection Kit (Lonza). Cells were detached with EDTA (2 mM, Invitrogen), and multicellular spheroids were generated according to the hanging drop method (Korff and Augustin, 1998). Briefly, hanging droplets of a 30 µl volume containing 5000 cells, methylcellulose (40% dissolved in DMEM; Sigma) and bovine collagen (10 µg/ml; Advanced Biomatrix) were incubated for 24 h to ensure multicellular aggregation. Spheroids were washed and resuspended in medium.

### Collagen–collagen interface assay

To prepare a spheroid–collagen culture, square silicon molds (Electron Microscopy Sciences) were attached to the bottom of a 24-well culture plate. Fibrillar high-density (6 mg/ml) or low-density (2 mg/ml) collagen gels were constituted by using rat tail collagen type I (Corning) supplemented with 10× Minimum Essential Serum (Sigma) and neutralized to a pH of 7.4 with 1 M NaOH, pipetted into the mold. Gels were polymerized at 37°C for 30 min in a humidified atmosphere, followed by addition of a single spheroid (where indicated), as well as medium (surface assay) or collagen solution (interface assay). For the latter, after addition of the spheroid, excess medium was carefully pipetted off, followed by addition of the collagen solution forming the top layer. Where indicated, GM6001 or DMSO were supplemented to collagen lattices as well as supernatant. Interface-containing collagen lattices without cells were fixed within 1 h after polymerization of the top gel, whereas spheroid-containing interface lattices were monitored within 1 h after top gel polymerization and further incubated for 48 h, after which they were fixed with 4% phosphate-buffered paraformaldehyde, washed and stained with the indicated antibodies and fluorescent dyes.

### Lattice cross-sectioning and microscopic imaging

Spheroid imaging was generally performed using high-content brightfield and epifluorescence microscopy combined with automated multi-position image acquisition and stitching (Leica DMI6000B; 10×/0.25 NA air objective). After *xy* imaging, gels were manually cross-sectioned from one side of the spheroid until the spheroid core was cut in the middle by using a scalpel during concomitant inspection under a dissection microscope (Leica MLFZIII). The lattice with the remaining half spheroid was then turned by 90° and imaged again over 24 *z*-scans in 10 µm intervals and overlaid for maximum *xz* image depiction of the spheroid (Fig. S3A). An exception to this was the image collection approach to compare *xz* views of the interface assay with the collagen surface assay (Fig. 2B), as the lattice generated for the surface assay was not stable enough to be cut, turned and imaged. Here, confocal microscopy (Zeiss LSM880; 10×/0.45 NA air objective) was used for image generation by *xy* imaging, *z*-sectioning with a distance of 3 µm between sections and image stitching. Confocal microscopy (Olympus FV1000; 40×/0.8 NA water objective) was used as well for the detection of collagen reflection and the collagen cleavage epitope. For high-resolution detection of the collagen interface by scanning electron microscopy (SEM), cell-free samples were fixed with 2% glutaraldehyde (Merck), washed and treated with 1% osmium tetroxide (Electron Microscopy Sciences). Samples were manually cross-sectioned, processed for SEM by dehydration through a graded series of ethanol followed by critical point drying using CO_2_, and then sputtered with gold and imaged (JSM-6340F, JEOL).

### Image analysis

Image processing and quantification was performed using Fiji ImageJ (1.52n; National Institutes of Health). Images were cropped, rotated, projected, manually adjusted for contrast and brightness, and displayed in virtual colors, from which readouts for guided interface migration (migration area, cell numbers, maximally migrated distance, thickness of interface migration zones) and non-guided 3D migration (migrated distance, cell numbers) were performed. For the calculation of the cell-populated area, images were processed by automated thresholding using the Triangle method, followed by filling of artificial holes (Fig. S2A,B). Cell numbers in the flat interface-migration area were quantified from a single slice after exclusion of the spheroid center, particle analysis using a median filter, automated thresholding (Default method) and watershedding of the DAPI signal, whereby areas larger than 1000 µm^2^ were excluded (Fig. S2D). The maximum migration distance was quantified by measuring the distance from the rim of the core to the six furthest cells and averaged per spheroid (Fig. S2F). The thickness of cell layers in the cleft and on the collagen surface was quantified by vertical *xz* display of the *xy* image stack (Fig. 2B). To quantify migrated cell numbers into either the cleft or the 3D collagen lattices, a region of interest (ROI) was drawn around the tumor that was expanded in steps of 50 µm or 25 µm, respectively (Figs S2D and S3B). For cells in the cleft and on the surface, the Fiji Cell Counter or Stardist plugins were used for automatic segmentation of nuclei (Zhao et al., 2022). For cells in 3D collagen lattices, the Fiji Cell Counter plugin was applied, and the number of cell nuclei between each two ROIs was counted manually and assigned to a zone when at least half of the cell nucleus was located in the area. From the counted cells in the 3D ECM zones, the sum total was corrected by a factor of 2.5 (Fig. S3D) because the imaged cross-sectioned lattice only contained half of the spheroid (thus 50%) and the imaging penetration depth was only ∼185 µm of the ∼230 µm depth (thus 80%). Lastly, the signal of the collagen 1 three-quarter fragment (Col1 ¾) was calculated as the mean gray signal from invasion areas, subtracted from background signal, and divided by the number of cell nuclei (Fig. 5A).

### *In silico* model

To predict the effects of collagen density and collagenolysis on cell migration, an *in silico* cellular automaton was developed (Deutsch and Dormann, 2018). The detailed mathematical procedures and simulation code were deposited at GitHub (https://github.com/TomPlt/Collagen-Interface-Model). Cell and ECM density of the experimental setup were represented by scalar fields defined on a 3D lattice with a lattice spacing of 10 μm. As an initial condition, we set a plane of low ECM density, representing the cleft, that was surrounded by high ECM density, and a spherical volume representing the high cell-density spheroid that was placed in the middle of the lattice (Fig. 4A panel I). Cell migration into the interface and porous 3D matrix was modeled as a deterministic rule that incorporated proliferation, ECM proteolysis and interface migration, and depended on the local microenvironment defined by six neighboring lattice sites (Von Neumann neighborhood; see Fig. 4A panel II, where four neighbors are shown). Proliferation was modeled as an increase in cell density according to the doubling time of MV3 cells. The assumption that cells were able to degrade surrounding ECM was implemented by a linear dependence of the ECM degradation rate on the cell density in the voxel and the neighboring voxels. For migration, two basic assumptions were made: (1) the surrounding ECM serves as a migration substrate and, when composed of linearly guiding low-density ECM regions, allows cells to adjust their movement direction (see increased cell flux during interface migration; Fig. 4A panel II); (2) cell migration is limited by available space and steric hindrance, which was implemented by a linearly decreasing relationship between outward migration rate and ECM density in neighboring voxels. This complex relationship between cell migration and the ECM neighborhood was implemented using a function with two regimes (see Fig. 4A panel III): (1) for low, increasing ECM densities in the near neighborhood, migration increases nearly linearly, with a maximum of this function being defined as the critical ECM density, a model parameter; and (2) for further increasing ECM densities in the neighborhood, migration speed decreases exponentially. Next, by matching the experimentally observed relative and absolute cell numbers in the interfaces for high- and low-density collagen in a parameter scan, the model parameters were identified that best describe the experiments (Table S1). The high 3D collagen density was accompanied by a low density between the layers (ECM_int_=0.1), while the low-density assay resulted in a less well-defined boundary with a relatively higher cleft density (ECM_int_=0.26), whereas the critical ECM density was always set at ECM_crit_=0.1. Thus, consistent with their relative differences in collagen density in the cleft versus 3D collagen (Fig. S1), a well-defined and a less precise shallower interface was found for the high- and low-density condition, respectively. To predict the effect of MMP inhibition on cell migration into the different compartments of the ECM, the density of the ECM interface was fixed at the mean value, and the 3D ECM density and degradation constants were systematically varied (Fig. 4C). Finally, the most likely combination of model parameters was identified for each experimental condition (Fig. 5J).

### Statistics

Statistical analysis was performed by the two-tailed unpaired Mann–Whitney test or *t*-test and, when necessary, by the Holm–Sidak method for correction of multiple testing using GraphPad Prism 8.3 software.

## Supplementary Material

10.1242/joces.260207_sup1 Supplementary information
