# Supplementary material for: Cell jamming in a collagen-based interface assay is tuned by collagen density and proteolysis
Source: J Cell Sci. 2023 Dec 15;136(23):jcs260207. doi: 10.1242/jcs.260207 (PMC10753497; doi:10.1242/jcs.260207)
Supplement: Supplementary information [file joces-136-260207-s1.pdf]

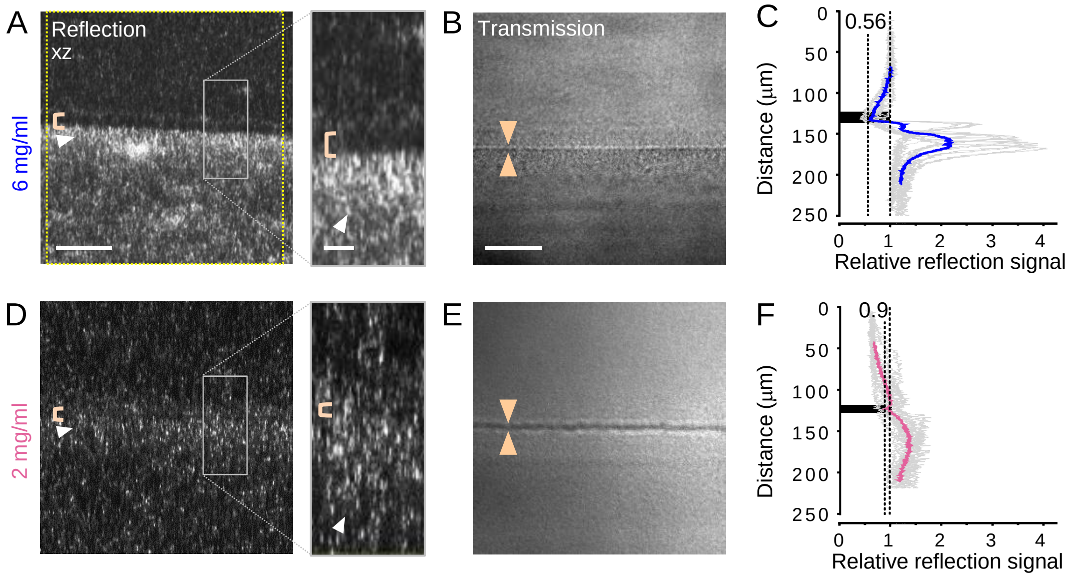

Fig. S1. Characterization of the collagen-collagen interface. Reflection-based analysis of collagen signal of manually cross-sectioned lattices. **(A,D)** Depiction of Interface-bordered clefts between each two collagen lattices of indicated concentrations. Arrowheads indicate regions of highest collagen density; braces indicate clefts. Reflection signal, side view. Yellow square indicates a 1500 pixel-wide area used for quantification in (C,F); red rectangles, regions of insets on the right**.** **(B,E)** Transmission signals indicating the cleft (arrowheads). **(C,F)** Calculation of cleft versus 3D matrix density ratios. Quantification of the mean gray values from top to bottom of image perpendicular to the interface [see approach in (A)], normalized to the intensities in porous 3D matrix and aligned along the lowest signal at the interface (see the position of the small rectangles in graded pastel color, which also indicate the approximate height of the respective clefts, based on the normalized signal <1). N=3; 4-5 images per experiment. Red lines, means; gray lines, individual experiments. Scale bars: 50 µm; inset: 10 µm.

Journal of Cell Science • Supplementary information

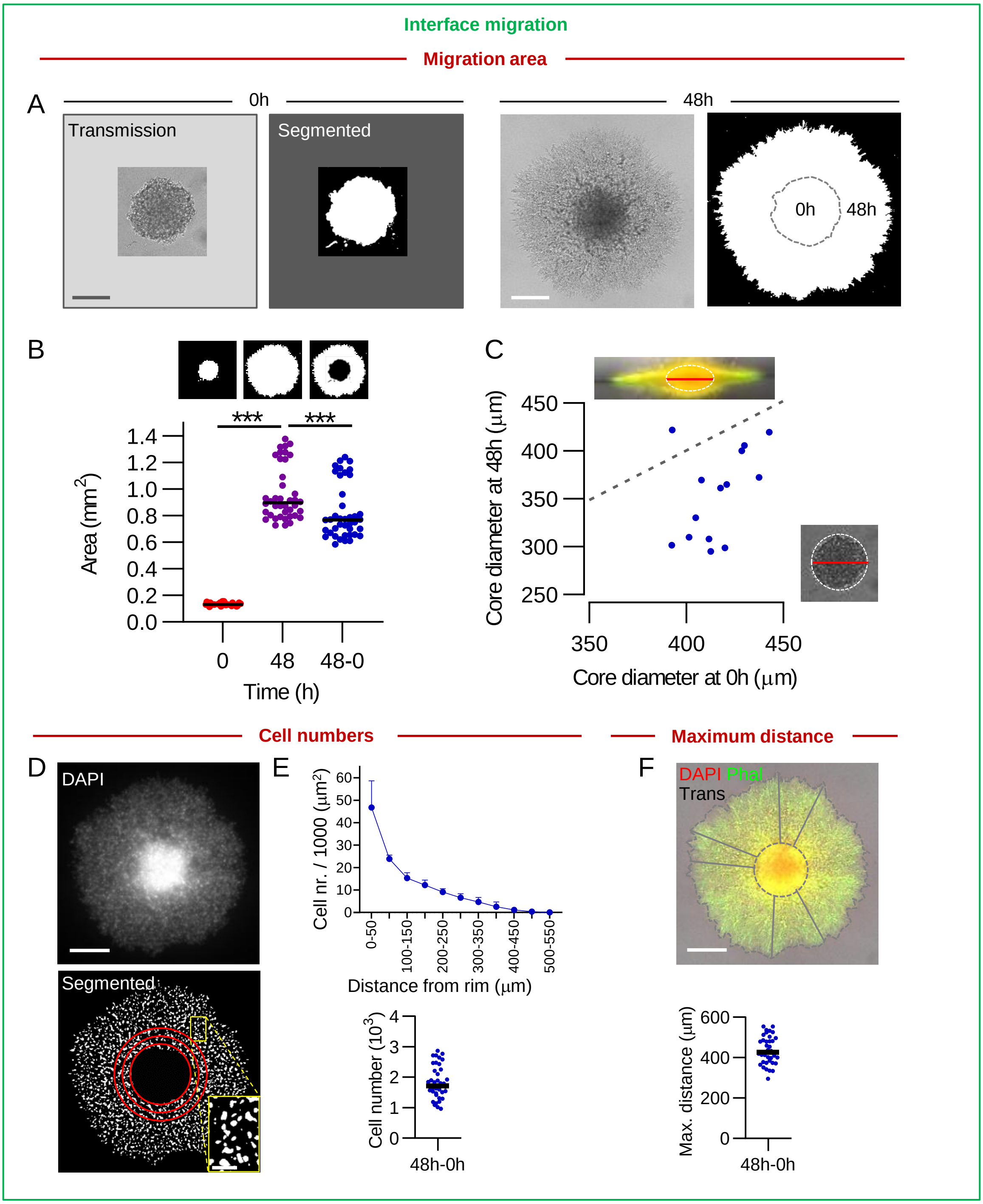

Journal of Cell Science • Supplementary information

Fig. S2. Approaches to quantify interface migration parameters 'Area', 'Cell numbers' and 'Maximum distance'. (A) Imaging of spheroids in the interface model (each left) and automated segmentation of the cell-populated area (each right) at 0h and 48h culture. **(B)** Quantification of segmented indicated areas. **(C)** Comparsion of spheroid core areas after 0 and 48h culture. Detected shrinkage of the spheroid core area after 48h culture (see gray diagonal line which indicates y=x; as well as circle and ellipse in Fig. 1G for visualization). **(D)** Automated segmentation of DAPI signal after manual exclusion of the core. Top, original microscopic image; bottom, corresponding segmentation and binning of regions into 50 μm sections. **(E)** Top, Quantification of cell numbers in relation to the distance from the rim. Bottom, Quantification of cell numbers away from the spheroid rim. **(F)** Top, Depiction of distance quantification from the spheroid rim to the six furthest migrated cell positions. Bottom, quantification of maximally migrated distance of cells away from the spheroid rim. **(E,F; lower rows)** Data represent 2-10 measurements per experiment (N=6). **(B,C,E,F)** Each dot represents a spheroid; solid line, median. Mann-Whitney test followed by Holm-Sidak; ***, $P < 0.001$. Scale bars: **(A,D,F)** 250 µm, **(D inset)** 50 µm.

Journal of Cell Science • Supplementary information

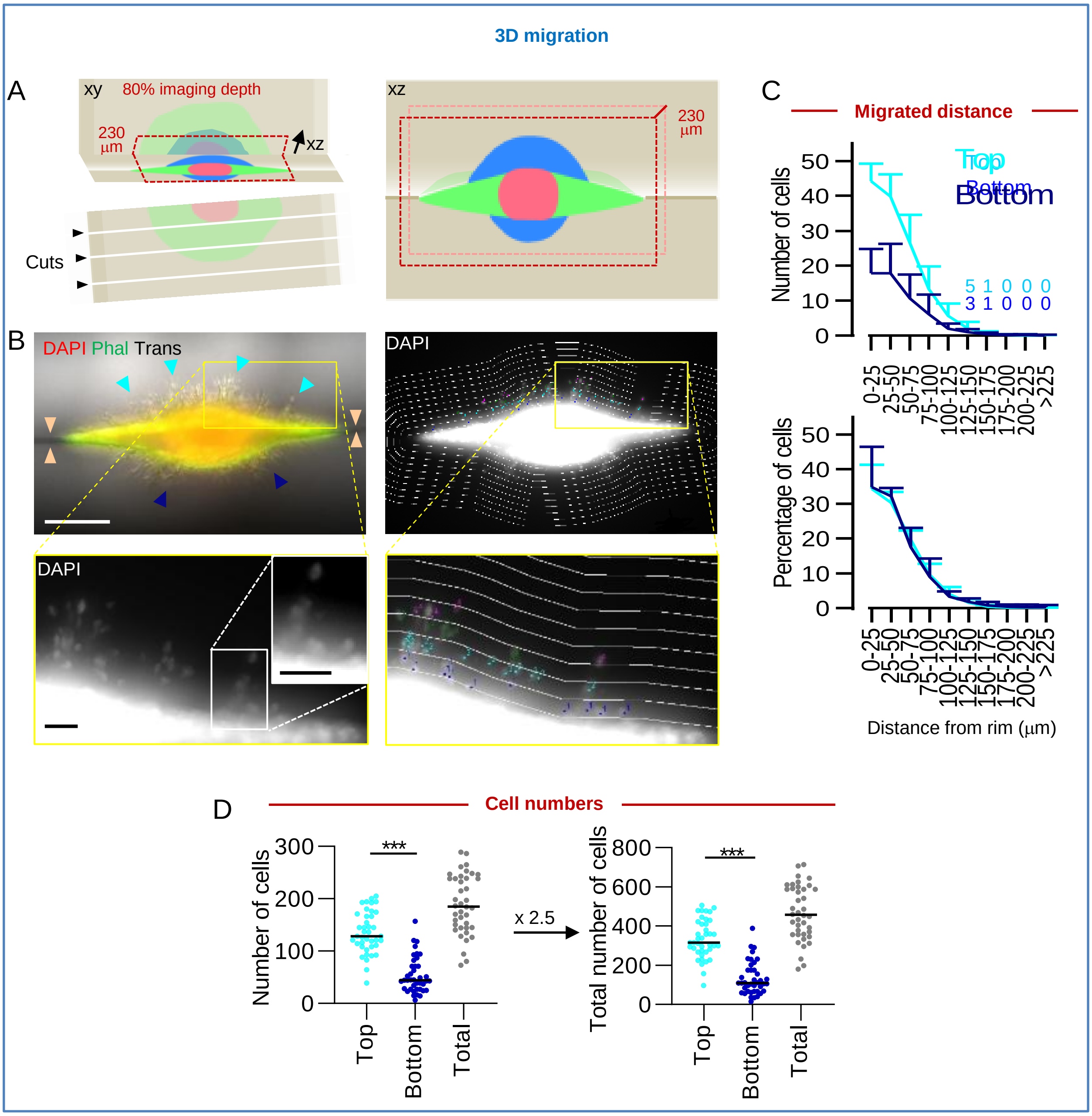

Journal of Cell Science • Supplementary information

Fig. S3. Approaches to quantify 3D migration parameters 'Distance' and 'Cell numbers'. 48h cell cultures were manually dissected to visualize and quantify cells that migrated into the 3D collagen lattices. **(A)** Cartoons depicting imaging strategy before and after manual cross-sectioning. **(B)** Left, cells migrating into dense 3D collagen (6 mg/ml) beyond the interface (upper lattice, light blue arrowheads; lower lattice, dark blue arrowheads; interfaces, orange arrowheads). Right, binning of 3D collagen regions into 25 µm sections (white dotted lines), created by expanding ROI's for counting of nuclei. Images are overlays of 24 z-stacks of each 10 µm distance, resulting in 230 µm deep widefield images as depicted in panel A. Colors represent cells in different ROI's. Bottom images are zoom-ins. **(C)** Quantification of cells located in the upper and lower 3D collagen lattices as a function of migrated distance from the rim of the spheroid core. Upper graph, absolute numbers of counted cells; numbers within the graph depict the cell counts that were located at the indicated distance from the spheroid rim. Lower graph, distribution of cells into the lower and upper collagen compartment. Data represent 2-10 spheroids per experiment (N=6). Solid line, mean; whiskers, SD. **(D)** Numbers of counted cells per top and bottom layers. Left, imaged 3D cell numbers; right, total 3D cell numbers. In all figures (3G,H; 5H,I,J; S4E), cells within the 3D collagen compartment were multiplied with the correction factor 2.5 (see Methods). Solid line, median. Each dot represents a spheroid. Mann-Whitney test, ****: $P < 0.0001$. Scale bars: 250 µm; insets: 50 µm.

Journal of Cell Science • Supplementary information

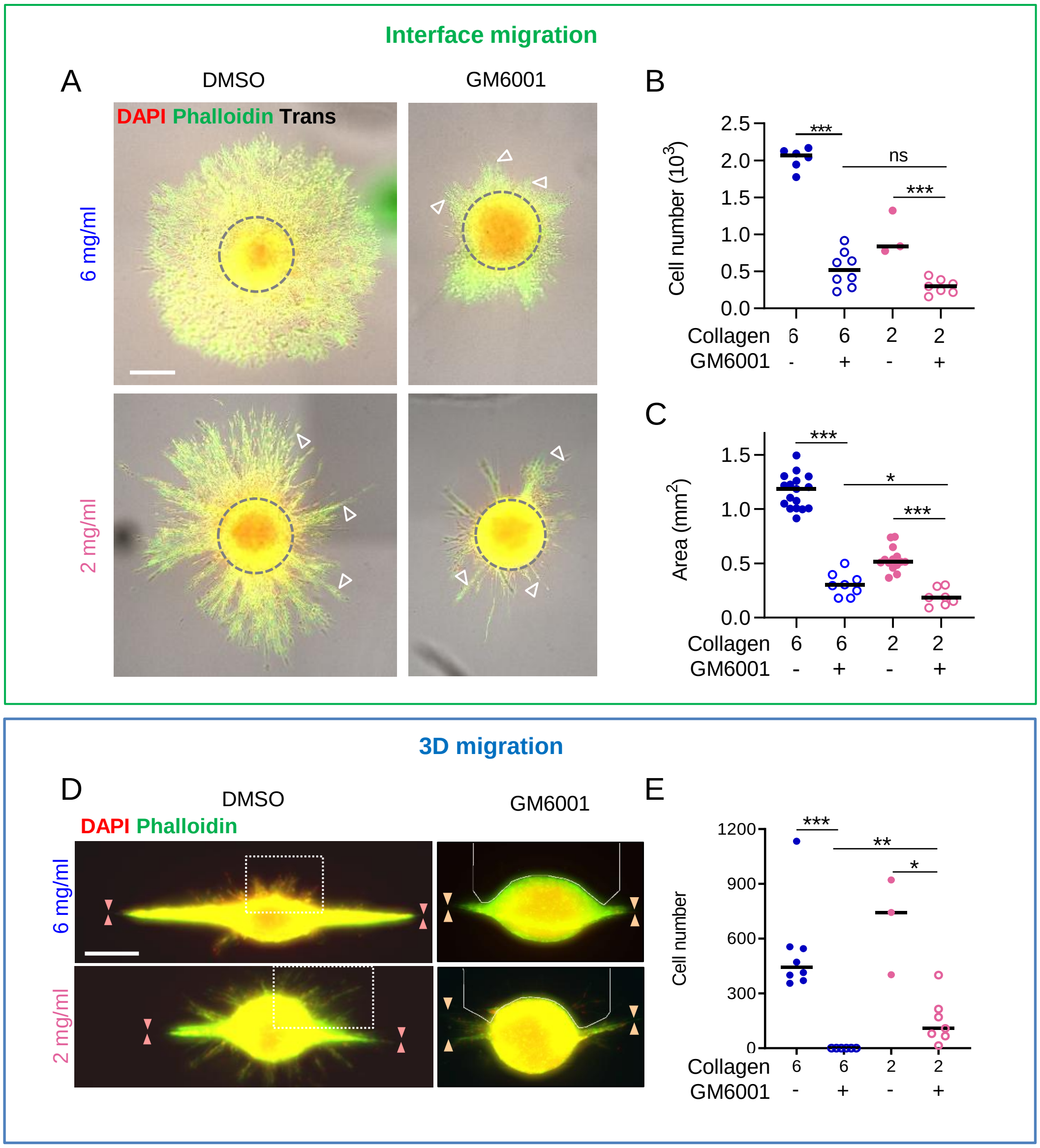

Fig. S4. Interface-guided- and 3D collagen migration depend on MMP-dependent collagen degradation. Images and data from Fig. 3. are here paired with the respective GM6001 conditions from Fig. 5 for better comparison. Depiction and quantification of cell emigration into the interface **(A-C)** or fibrillar collagen **(D,E)** in the absence or presence of GM6001. **(A)** Empty arrowheads indicate strand-like migration **(A)**. **(B,C,E)** Each dot represents a spheroid, median, solid line. Mann-Whitney test followed by Holm-Sidak test; ns, not significant; *, $P < 0.05$; **, $P < 0.01$; ***, $P < 0.001$. All scale bars: 250 µm.

Journal of Cell Science • Supplementary information

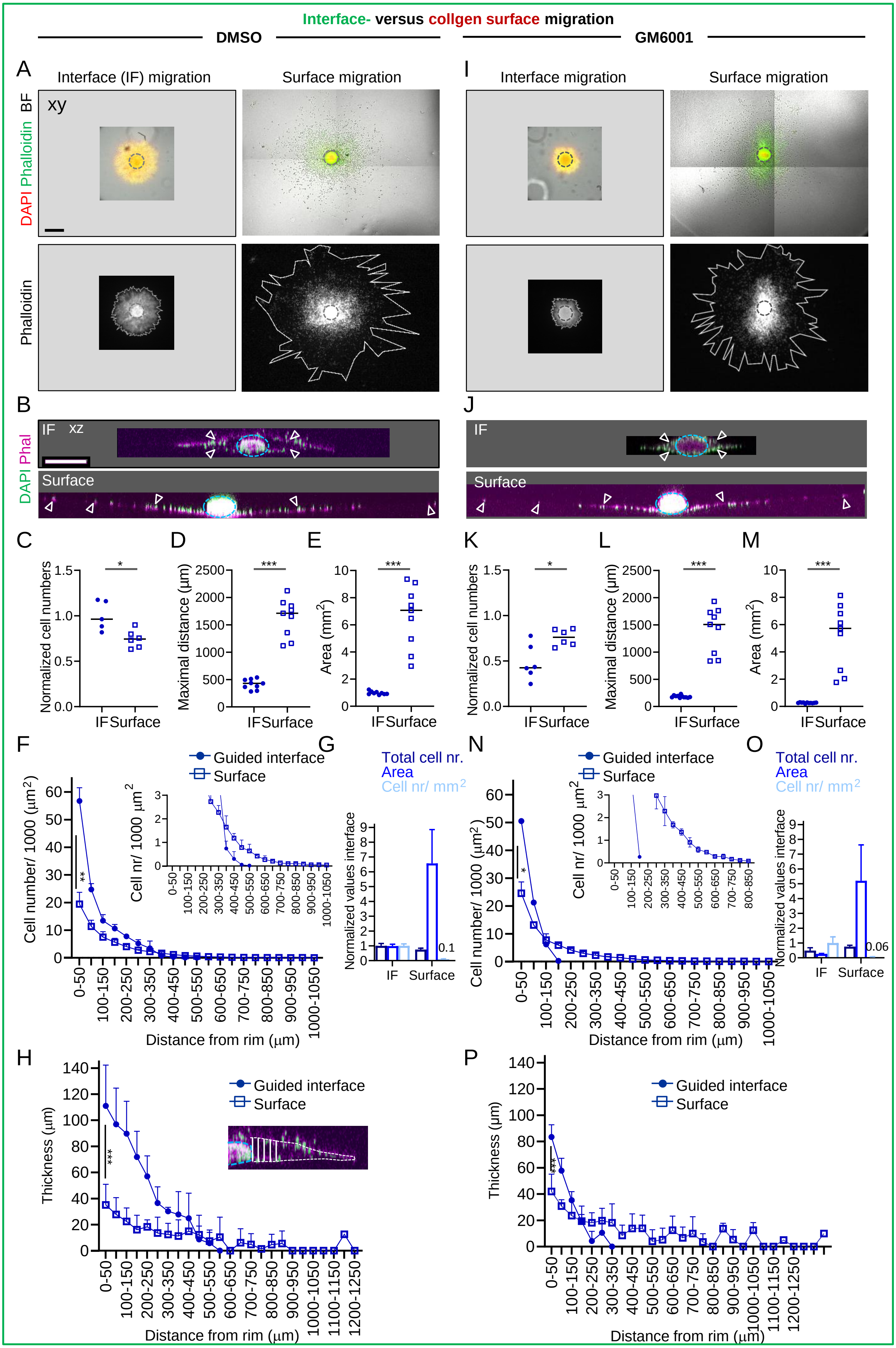

Journal of Cell Science • Supplementary information

Fig. S5. MV3 melanoma cell migration along the interface and over a 3D collagen gel surface in the absence or presence of GM6001. For better comparison, images and data from Fig. 2 (here **A-H**) are paired with data in the presence of GM6001 **(I-P)**. For description of the individual panels, please see Fig. 2 legend. The difference for guided interface migration (at 0-50 mm distance from rim) in panel H and P was significant (unpaired t-test; *, $P < 0.05$).

Table S1. Model parameter values after fitting the *in silico* cellular automaton model for the experimentally observed migration numbers in the collagen lattices of both indicated concentrations.

Collagen concentration	Interface density	Mean interface density	Porous 3D ECM density	Critical density	Degradation constant	Mean degradation constant
**6 mg/ml**	[0.08, 0.12]	[0.1]	[0.74, 1.00]	0.1	[0.12, 0.18]	[0.15]
**2 mg/ml**	[0.2, 0.32]	[0.26]	[0.46, 0.52]	0.1	[0.04, 0.08]	[0.06]

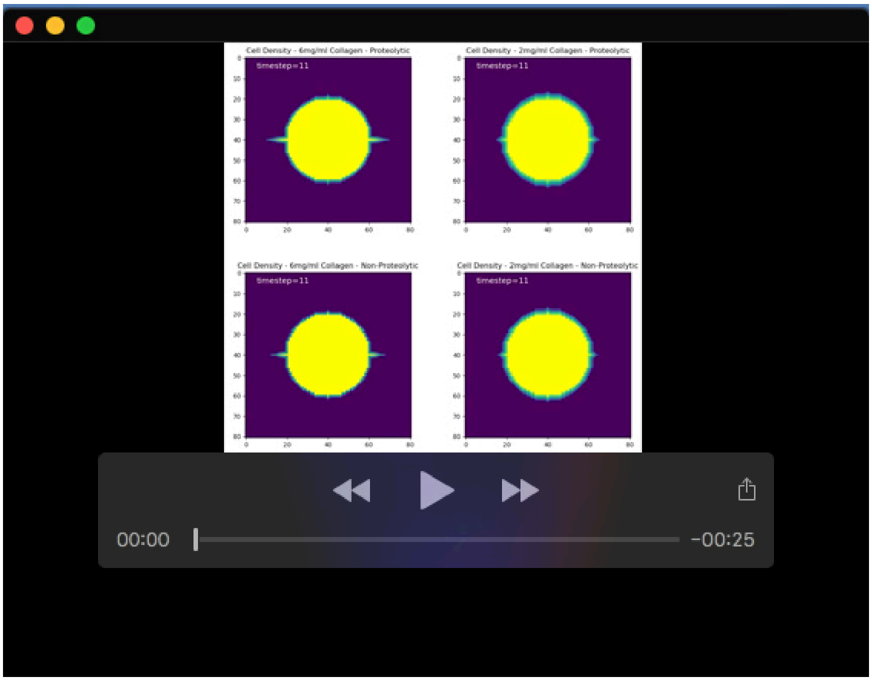

Movie 1. Depiction of cell density over 100 time steps at varying collagen density (from left to right) and collagen degradation values (from top to bottom) generated by the cellular automaton model. Movie corresponds to data shown in Fig. 4B and to the color scheme shown in Fig. 4a.

Journal of Cell Science • Supplementary information
