# Supplementary material for: Cell jamming in a collagen-based interface assay is tuned by collagen density and proteolysis
Source: J Cell Sci. 2023 Dec 15;136(23):jcs260207. doi: 10.1242/jcs.260207 (PMC10753497; doi:10.1242/jcs.260207)
Supplement: Supplementary file 1 [file joces-136-260207_review_history.pdf]

Cell jamming in a collagen-based interface assay is tuned by collagen density and proteolysis

Lianne Beunk, Nan Wen, Sjoerd van Helvert, Bram Bekker, Lars Ran, Ross Kang, Tom Paulat, Simon Syga, Andreas Deutsch, Peter Friedl and Katarina Wolf
DOI: 10.1242/jcs.260207

Editor: Kathleen Green

Review timeline

Original submission:	1 July 2022
Editorial decision:	23 August 2022
First revision received:	31 October 2023
Accepted:	15 November 2023

Original submission

First decision letter

MS ID#: JOCES/2022/260207

MS TITLE: Extracellular matrix guidance determines proteolytic and non-proteolytic cancer cell patterning

AUTHORS: Lianne Beunk, Sjoerd van Helvert, Bram Bekker, Lars Ran, Ross Kang, Tom Paulat, Simon Syga, Andreas Deutsch, Peter Friedl, and Katarina Wolf
ARTICLE TYPE: Tools and Resources

We have now reached a decision on the above manuscript.

To see the reviewers' reports and a copy of this decision letter, please go to: https://submit-jcs.biologists.org and click on the 'Manuscripts with Decisions' queue in the Author Area. (Corresponding author only has access to reviews.)

As you will see, the reviewers raise a number of substantial criticisms that prevent me from accepting the paper at this stage. Their enthusiasm for the extent to which the work advances the field differs somewhat, with referee #3 stating:

"While this manuscript presents a methodology to tune the interface, it is somewhat incremental and the results are fairly intuitive. So while interesting, the advance in the field is not as significant."

However, based on a greater degree of enthusiasm by other referees, I would be amenable to considering a revised version that addresses their technical comments, and makes an argument for why the work will add significantly to the tools available to the field.

We would then return it to the reviewers.

Please ensure that you clearly highlight all changes made in the revised manuscript. Please avoid using 'Tracked changes' in Word files as these are lost in PDF conversion.

© 2023. Published by The Company of Biologists under the terms of the Creative Commons Attribution License (https://creativecommons.org/licenses/by/4.0/).

I should be grateful if you would also provide a point-by-point response detailing how you have dealt with the points raised by the reviewers in the 'Response to Reviewers' box. Please attend to all of the reviewers' comments. If you do not agree with any of their criticisms or suggestions please explain clearly why this is so.

Reviewer 1

Advance summary and potential significance to field

In this manuscript, the authors address an interesting question in cancer biology involving the behaviour of tumour cells in clefts or interfaces in vivo. They develop a new assay in which a layer of collagen is polymerized first, then next a melanoma cell spheroid is positioned on this lower gel, and then a gel of the same concentration is poured and polymerized above it to produce to collagen layers with an interface of differing collagen. The authors find that the interface between these two gel layers has a significantly lower concentration of collagen with fewer fibrils. This interface or cleft is most apparent between 6 mg/ml high concentration collagen gels compared to 2 mg/ml collagen gels. The authors then quantify the expansion of cells from the spheroid, which is much greater along the interface than into the 3D gels facing each other. They provide evidence that MMP based proteolysis is most effective against 3D gel migration compared to interface migration, which they suggest may be relevant clinically in the sense that tumours invading in clefts or interfaces may be resistant to protease inhibitors. The experimental work is complemented by a computational model, which supports the findings.
The general question in cancer biology addressed by the authors involving the propensity of cells to invade along clefts or interstices and how this property can be elucidated is interesting and important. The assay described by the authors is potentially clever and interesting as a possible means of addressing these types of questions. The results presented seem to be of generally high quality and the illustrations are often excellent. Although the results presented are consistent with the conclusions presented, there are major questions that need to be answered about this assay and most importantly, whether the assay is evaluating translocation of the cancer cells along interfaces as opposed to merely migrating in a zone of low porosity.

Comments for the author

Major questions:

1. A major concern is why the bottom gel has so much higher reflectance and even differences in porosity according to SEM. If polymerized and allowed to sit for significant amounts of time, it could begin to dry out and generate higher density. The lower surface may then become a substrate (see next point). The authors need to provide details about the timing of the steps in their assay to possibly lessen this concern.

2. It is not clear whether the main findings of this assay are merely due to the fact that the bottom gel becomes a substrate for better cell migration. This point becomes significant with the GM6001 differences of cell behavior at the interface compared to migration up or down into the main gels. The most important control experiment will be to culture the spheroid on the bottom gel with no top gel in the presence or absence of GM6001 to elucidate whether one obtains the same results as in the interface model, just due to providing a flat, favorable migration surface.

3. It needs to be clarified whether the phenomena observed are due to an interface or instead due to the lower porosity. A control for this possibility is to compare the collective cell invasion from spheroids polymerized inside a uniform collagen gel with an appearance matching the same porosity and concentration as the interface, with and without protease inhibitors. For example, if the pore sizes are large, the same results with respect to more resistance to protease inhibition would likely be obtained compared to a uniform high percentage gel of low porosity. That is, a control experiment for the latter concern is to treat spheroids in the middle of 6 mg/ml collagen compared to spheroids within a 1-2 mg/ml gel to see if the same differences are observed due to the porosity rather than the interface.

4. The results shown in the in silico cellular automata Figure 3 were very difficult for this reviewer to comprehend. The use of different colour schemes i.e., grayscale compared to the same colour

© 2023. Published by The Company of Biologists under the terms of the Creative Commons Attribution License (https://creativecommons.org/licenses/by/4.0/).

scheme for both ECM density and Cell density was confusing. This reviewer could not decipher the panel labeled C!,
Either a simpler or more understandable scheme would be needed, or the authors would need to provide an appendix to explain in detail what it is that they wish to show. It was quite difficult to see how the results from this modeling support what was observed, since the results were not nearly as clear in this model as shown for in vitro.

5. The weakness of in silico modeling is that the modeler can often adjust parameters to fit any results obtained from experiments. The great value of such computational modeling is the ability to make testable predictions, ideally of findings that are not intuitively obvious. It would be most reassuring if the authors could use their model to make such a prediction and to test it.

6. The magnitudes of differences in interface migration with GM6001 treatment reported are not large, since Figure 4J has cut off 50% of the scale. They are nevertheless significant, but one is concerned that that result was due to the issue in point #3. Additionally, the inhibition by GM6001 of interface cell number is quite strong, i.e., 80% in Supplementary Figure 4B. Is this really “MMP-independent migration”?

Less important:

7. There seems to be increased density of collagen fibrils adjacent to the interface that is higher than the main collagen gel itself, e.g., in Figures 1B 1C, Suppl. Figure 1A and 1E. Does this increased density affect cell behaviour e.g., by forming a stiffer substrate?

8. This reviewer could not understand Figure 4 I, where the symbol legend for Control and GM-6001 and “Expe-riment” did not seem to make sense. What is the weak red-orange dashed line near the x-axis in the left graph mean? Might there have been a problem with producing the PDF for this figure?

9. It was not sufficiently clear what was meant by “matrix guidance” in this paper. At one point in the modeling, contact guidance is mentioned, but that is often a manner of migration following linear fibers or grooves. Elsewhere, the paper refers to a low density guiding interface, but is this just a narrow zone of low concentration collagen between two high concentration gels? Basically, what exactly is the mechanism of guidance, or is the term just used generally?

Very minor:

19. It would have ideal for the authors to test more than one cell type especially a "normal" cell to evaluate whether the findings are specific to cancer.

20. On page 9, the text refers to “a number of around 2000” Is the word “cells” missing?

It is hoped that the authors will be able to clarify these questions and resolve the concerns about this seemingly clever new assay.

<u>Reviewer 2</u>

Advance summary and potential significance to field

The authors developed a new method based on a 3D type I collagen assay containing a cleft-like interface between two lattices of collagen. They then studied the invasion of cancer spheroids into the gel. The interface has a higher porosity (authors claimed 2-10x increase which is quite heterogeneous). Based on their results and characterization, the authors claimed that this interface is Â“low-density guiding interface to disentagle the relative impact of ECM-derived barriers versus guidance cuesÂ”. It appears that the guidance cues are of two kinds: a decrease of porosity at the interface but also a confinement between 2 layers of denser collagen gels. The main novelty of the paper relies on the development of a simple assay to guide cell invasion and migration.

Comments for the author

© 2023. Published by The Company of Biologists under the terms of the Creative Commons Attribution License (https://creativecommons.org/licenses/by/4.0/).

The authors developed a new method based on a 3D type I collagen assay containing a cleft-like interface between two lattices of collagen. They then studied the invasion of cancer spheroids into the gel. The interface has a higher porosity (authors claimed 2-10x increase which is quite heterogeneous). Based on their results and characterization, the authors claimed that this interface is “low-density guiding interface to disentagle the relative impact of ECM-derived barriers versus guidance cues”. It appears that the guidance cues are of two kinds: a decrease of porosity at the interface but also a confinement between 2 layers of denser collagen gels. The main novelty of the paper relies on the development of a simple assay to guide cell invasion and migration. The biological results are not new and supported by previous papers published by Friedl and Wolf’s group and other studies. Overall, I think that the paper may be of interest for the cell biology community and I could recommend its publication after some revisions.

Comments:
1/ SEM should be provided for both conditions (interface between dense 6mg/ml and less dense 2mg/ml collagen) and analyze pores sizes in these conditions. This is an important point to disentangle the impact of pore size and high/low-density ECM.
2/ Concerning the inhibitor, I am wondering if the confinement of the cells could trigger the sensitivity to GM6001 ?
What would be the effect of GM6001 on cell invasion in homogeneous large pore or small pores of 2mg/ml or 6mg/ml? Would migration patterns be the same (collective versus tracks)?
3/The authors claimed that increased single cell invasion is observed at lower densities. It does not seem to be obvious from the current data. This point should be clarified.
4/ For the modeling part, I think that the implementation of matrix degradation should be clarified. Furthermore, the authors claimed that they “identified the high 3D collegen density to be …” (P11 middle). I do not understand since it is implemented in the model and could not be an output. Further details are thus required.
5/ The authors claimed that they observed subcellular protrusions (p12 middle) of great length (Fig 4B, E). I had some difficulty to observe them. Could they provide better images?

<u>Reviewer 3</u>

Advance summary and potential significance to field

In this manuscript, the authors present a model of spheroid migration at an interface of collagen. The interface presents the cells with a choice of dense or less dense(a "cleft") collagen in which to move. The authors then parse apart where cells move depending on the porosity and application of protease inhibitors.
There has been significant work done in the area of proteolytic vs non-proteolytic migration in the field, by this group and others, and there has been work done previously investigating the behavior of cells at interfaces. While this manuscript presents a methodology to tune the interface, it is somewhat incremental and the results are fairly intuitive. So while interesting, the advance in the field is not as significant. The value of the model could be better demonstrated if some new biology is learned.

Comments for the author

The study is fairly straightforward. I have just minor comments:

Figure 1E: Authors state the cells spread to 6times the spheroid, however this value only has meaning when compared to the time allowed for spreading. Also, it is not clear what they mean by the spheroid not changing. Based on the images provided it is not possible to tell what the geometry or size of the spheroid core is compared to the cells migrating out from it. It appears to be expanding but it not clear how one defines the core.

Figure 1F needs better labeling. Do the white areas indicate cuts?

In general, the results should be reported as a function of time since all the differences in migration are measured for a given time period.

© 2023. Published by The Company of Biologists under the terms of the Creative Commons Attribution License (https://creativecommons.org/licenses/by/4.0/).

A control should be run to ensure the inhibitors are working as desired (ie able to diffuse sufficiently to inhibit proteases as desired).

First revision

Author response to reviewers' comments

Our response letter to the reviewers is long and contains some figures. In order to not loose the formatting, we attach our reviewer responses as a separate pdf.

Rebuttal letter (Beunk et al.)

Below we have addressed all concerns raised by the three reviewers. As a general remark, text additions that add new content to the manuscript are highlighted in ***bold/italic*** in this rebuttal letter, and in brown in the manuscript. We have further briefly introduced the term ‘cell jamming’, now used throughout the manuscript and added content requested in the authors checklist, highlighted as well. In a limited number of cases, we have removed or moved sentences, took out redundancies, or specified content for better flow, which improved wording but has not changed the overall content of the manuscript and therefore was not highlighted. Last, we exchanged the somewhat misleading term ‘random collagen’ with ‘porous collagen’ or ‘3D collagen’, and made more specific use of the terms ‘cleft, cleft boundaries and interface’.

Reviewer 1 Advance Summary and Potential Significance to Field:
In this manuscript, the authors address an interesting question in cancer biology involving the behaviour of tumour cells in clefts or interfaces in vivo. They develop a new assay in which a layer of collagen is polymerized first, then next a melanoma cell spheroid is positioned on this lower gel, and then a gel of the same concentration is poured and polymerized above it to produce to collagen layers with an interface of differing collagen. The authors find that the interface between these two gel layers has a significantly lower concentration of collagen with fewer fibrils. This interface or cleft is most apparent between 6 mg/ml high concentration collagen gels compared to 2 mg/ml collagen gels. The authors then quantify the expansion of cells from the spheroid, which is much greater along the interface than into the 3D gels facing each other. They provide evidence that MMP based proteolysis is most effective against 3D gel migration compared to interface migration, which they suggest may be relevant clinically in the sense that tumours invading in clefts or interfaces may be resistant to protease inhibitors. The experimental work is complemented by a computational model, which supports the findings.
The general question in cancer biology addressed by the authors involving the propensity of cells to invade along clefts or interstices and how this property can be elucidated is interesting and important. The assay described by the authors is potentially clever and interesting as a possible means of addressing these types of questions. The results presented seem to be of generally high quality and the illustrations are often excellent. Although the results presented are consistent with the conclusions presented, there are major questions that need to be answered about this assay and most importantly, whether the assay is evaluating translocation of the cancer cells along interfaces as opposed to merely migrating in a zone of low porosity.

We appreciate this generally favorable feedback on the assay and the quality of the results and the imaging. We further appreciate the reviewer’s constructive criticism, which we address point by point below.

Comments for the Author: Major questions:
1. A major concern is why the bottom gel has so much higher reflectance and even differences in porosity according to SEM. If polymerized and allowed to sit for significant amounts of time, it could begin to dry out and generate higher density. The lower surface may then become a substrate (see next point). The authors need to provide details about the timing of the steps in their assay to possibly lessen this concern.

© 2023. Published by The Company of Biologists under the terms of the Creative Commons Attribution License (https://creativecommons.org/licenses/by/4.0/).

After polymerization of collagen hydrogels over 30 min in the humidified environment of the incubator and with the lid on the plate kept closed, significant evaporation is unlikely. We have revised the methods description for clarity, as follows (page 12): ***'Gels were polymerized at 37°C for 30 min in humidified atmosphere, followed by addition of a single spheroid (where indicated), as well as medium (surface assay) or collagen solution (interface assay). For the latter, after addition of the spheroid excess medium was carefully pipetted off, followed by addition of the collagen solution forming the top layer.'.***

In addition, we sought to better understand the difference of reflectance intensity between lower and upper layer of the interface. We noticed that the dense layer on the bottom gel only occured when a top collagen layer was added. Adding medium at different amounts (Figure R1a, for the reviewers' discretion) did not induce this compacted layer. To also address whether gravity contributed to this heterogeneity, we flipped the collagen droplet during polymerization. We reasoned that inversing gravity could avoid potential collapse and compaction of the emerging collagen polymer interface in conjunction with adding a second layer. Despite this inverted approach, the difference of reflectance intensity was reproducible (Figure R1b, for the reviewers' discretion). We thus reason that adding weight from a second collagen layer is sufficient to induce bottom gel densification, and consider this phenomenon intrinsic to the generation of our interface collagen model. We have added this phrase to the Results section (page 5): '... ***assuming compression by the weight of the top gel but not*** ...'. If this reviewer wishes, we will add Figure R1a (or parts of it) to this manuscript. We have further added this phrase to the Discussion section on page 9: '***presumably induced by adding weight from a second collagen layer, a phenomenon that we consider intrinsic to the generation of our interface collagen model.***'

To better represent the cleft geometry and collagen compaction zone along the cleft, which was highly reproducible, we have updated Figure S1A,D. We accordingly revised the related data sections at page 5: ***'The bottom and top lattices formed two interfaces with a cleft in between them that was initially around 10 µm in height and consisted of bridging collagen fibrils at low density, together also named 'interface' (Figs. 1B,C; S1A,B). The densities of the interface boundaries which guide and confine cells were asymmetric, with high density downward. The upper gel, when compared to the cleft, consisted of an up to 45% gradually increasing signal intensity over a 50 µm wide fiber network until it merged with the intensity of the top layer (Fig. S1C). The collagen-compacted top layer of the bottom gel, when compared to the cleft, consisted of an 3 to 4-fold increased signal, and the remaining bottom gel of an about 2-fold increased reflection signal (Figs. 1B; S1A,C).'***; page 5: ***'Decreasing the concentration to 2 mg/ml resulted in a less prominent cleft, characterized by a mildly reduced height (5-8 µm), with only around 10% difference in signal intensity between cleft and the upper 3D matrix'***; and page 9: ***'Migration in our in vitro interface assay occurred predominantly along 5-10 µm high clefts of low ECM density bordered by high-density matrix, reminescent to largely linear interfaces and clefts in insterstitial tissue in vivo (…).'***

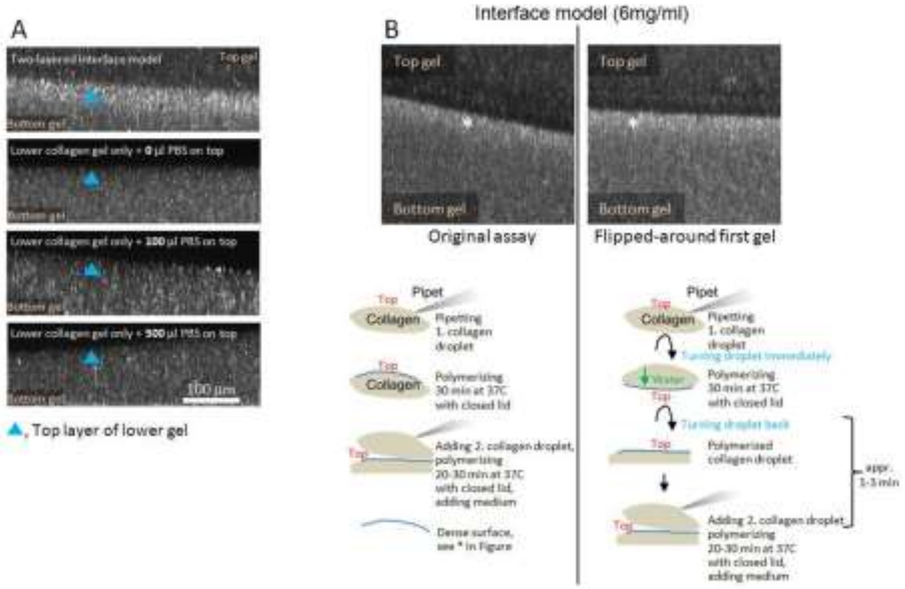

Figure R1.

© 2023. Published by The Company of Biologists under the terms of the Creative Commons Attribution License (https://creativecommons.org/licenses/by/4.0/).

2. It is not clear whether the main findings of this assay are merely due to the fact that the bottom gel becomes a substrate for better cell migration. This point becomes significant with the GM6001 differences of cell behavior at the interface compared to migration up or down into the main gels. The most important control experiment will be to culture the spheroid on the bottom gel with no top gel in the presence or absence of GM6001 to elucidate whether one obtains the same results as in the interface model, just due to providing a flat, favorable migration surface.

We appreciate this insightful suggestion and allowed cells to move across the collagen gel surface without additional upper layer in the absence or presence of GM6001 (new Figs. 2, S5). Cells sheets emigrating from spheroids across an unconfined collagen surface covered a seven-fold increased area compared to 3D interface conditions. Notably, the cell numbers emigrating in 2D unconfined vs. 3D confined cleft conditions were similar (Fig. 2C). This indicates that the interface, besides providing guidance, also mechanically confines the moving cell sheet which delays outward migration and, in addition, favors transition to multi-layered organization which can be appreciated from the the side view (see Fig. S4D, upper left image; Figs. S5B,H). This indicates that the interface provides both guidance and mechanical resistance and thereby induces cell jamming, similar to preformed laser tracks [1] and 3D clefts naturally occurring in normal interstitial tissue [2] and peri-tumor stroma [3] .. We have included this into the discussionon page 9 as '***In vitro and in silico data show that the presence of a low-density cleft provides both guidance and mechanical resistance and thereby induces cell jamming, to (1) support cell motility and (2) enable MMP-independent migration by cell jamming***.'. We have further described the new methodology in the Method part accordingly at 'Lattice cross-sectioning' and 'image analysis' at page 13: '***An exception formed the image collection approach to compare XZ views of the interface assay with the collagen surface assay (Figs. 2B), as the lattice generated for the surface assay was not stable enough to be cut, turned and imaged. Here, confocal microscopy (Zeiss LSM880, 10x / 0.45 NA air objective) was used for image generation by xy imaging, z-sectioning with a distance of each 3 µm, and stitching. Confocal microscopy (Olympus FV1000, 40x / 0.8 NA water objective) was used as well for the detection of collagen reflection, and collagen cleavage epitope***.' and page 13: '***The thickness of cell layers into cleft and onto collagen surface*** *was quantified by vertical xz display of the xy-image stack (Fig. 2B). To quantify migrated cell numbers into either the cleft or the 3D collagen lattices, a region of interest (ROI) was drawn around the tumor that was expanded by each 50µm* ***or 25 µm, respectively (Figs. S2D and S3B). For cells in the cleft and on the surface, the Fiji Cell Counter- or Stardist plugins were used for automatic nuclei segmentation [4]. For cells in 3D collagen lattices, the Fiji Cell Counter plugin was applied, the number of cell nuclei between each 2 ROI's was counted manually and assigned to a zone when at least half of the cell nucleus was located in the area.***'. In addition, we have described these new results as follows (page 6): '***Cell migration along 3D clefts results in a jamming-like phenotype. To address whether the effective migration into the interface was caused by specifics of the 3D cleft, including, e.g., by the dual-contact with the upper and lower interface, we performed control experiments seeding spheroids on top of a high-density collagen gel without additional collagen layer. In this 2.5D-like setting, cells on a collagen gel surface migrated both as bulk and as single cells at similar numbers compared to invasion into the cleft, but covered up to 3.5-fold increased distance, represented as a 7-fold larger area coverage compared to interface migration (Fig. 2A, C-F). As a consequence of 3D confinement, we observed a transition to multilayered sheet organization of around 3-fold increased thickness and with an overall 10-fold increased cell density, whereas unconfined migration across the collagen surface yielded in majority a monolayer (Fig. 2B, F-H).***
Together, the interface in a high-density matrix environment guides migration of large cell numbers into limited space, leading to cell jamming.'.
In the presence of GM6001, the number of invading cells was reduced by 50-75% in the interface assay and undiminished in the surface assay (Figs. S4B; S5C,K). This indicates that invasion into the cleft is, in part, favored by but not completely dependent on MMP-mediated proteolytic remodeling, in contrast to invasion into the randomly organized high-density 3D compartment, which fully depends on MMP activity. Please also see our answer to point 6 of this reviewer. In summary, the interface model depicts a migration assay to model the extent of cell jamming, to

© 2023. Published by The Company of Biologists under the terms of the Creative Commons Attribution License (https://creativecommons.org/licenses/by/4.0/).

be used for different inteference conditions. Further, throughout the manuscript, we use the term “confined migration”, which denotes high cell density and a jamming transition to collective movement of mesenchymal cells, as observed and described for the migration of proteolytic tumor cells into high- density matrix previously [5, 6]. In the introduction (page 3) we state: ‘... ***As a result of the latter, barrier-free tracks are generated that laterally confine and guide cells, but also gradually widen and become filled by follower cells, leading to the phenomenon of cell jamming’***

3. It needs to be clarified whether the phenomena observed are due to an interface or instead due to the lower porosity. A control for this possibility is to compare the collective cell invasion from spheroids polymerized inside a uniform collagen gel with an appearance matching the same porosity and concentration as the interface, with and without protease inhibitors. For example, if the pore sizes are large, the same results with respect to more resistance to protease inhibition would likely be obtained compared to a uniform high percentage gel of low porosity. That is, a control experiment for the latter concern is to treat spheroids in the middle of 6 mg/ml collagen compared to spheroids within a 1-2 mg/ml gel to see if the same differences are observed due to the porosity rather than the interface.

We have previously shown that MMP-independent migration efficacy declines with increasing collagen density [6, 7]. The fact that protease-dependent 3D ECM migration in our assay was partly inhibited in 2 mg/ml assays, but completely in 6 mg/ml assays confirms these previous data. The finding that MMP-independent migration in the cleft was partially reduced (by 50-75%) inhibited in both low- and high-density gel conditions (2 and 6 mg/ml; see Fig. S4B) may suggest that the level of confinement corresponds to a pore size of 8-20 μm^2 in hydrated interface-free collagen gels (see Reference [7], and Figs. 1D-F; Fig. 2 therein).
Unfortunately, here we could not measure the actual “pore size” in the cleft in a meaningful manner. Electron-microscopically- derived collagen gel images (Fig. 1C) do not reflect the true pore size, as these gels shrink during sample processing. The axial resolution of confocal reflectance microscopy is insufficient to address the pores between bridging fibres in collapsed, cell-free clefts. And with cells invading along the cleft, the presence of the cell body precludes reliable detection of the space between collagen fibrils. Thus, we decided not to include such measurements and hope this reviewer will agree.
To address the issue of bridging collagen fibrils, we state in the Results (page 5 and stated above): ‘***The bottom and top lattices formed two interfaces with a cleft in between them that was initially around 10 µm in height and consisted of bridging collagen fibrils at low density, together also named ‘interface’ (Figs. 1B,C; S1A,B).***’, and in the Discussion (page 9): ***‘Our electron-microscopically-derived imaging further reveals bridging collagen fibrils within the cleft, with an estimated 2- 10 times increased pore size range when compared to the surrounding 3D collagen (Fig. 1C). Even though precise pore size measurements are not possible as such collagen gel images shrink during sample processing, and the axial resolution of confocal reflectance microscopy is insufficient to address the pores in collapsed cell-free clefts, cell behaviour in the presecne of GM6001 indicates that these pore sizes might be between 8-20 μm^2 large.’.***

4. The results shown in the in silico cellular automata Figure 3 were very difficult for this reviewer to comprehend. The use of different colour schemes, i.e., grayscale compared to the same colour scheme for both ECM density and Cell density was confusing. This reviewer could not decipher the panel labeled C!, Either a simpler or more understandable scheme would be needed, or the authors would need to provide an appendix to explain in detail what it is that they wish to show. It was quite difficult to see how the results from this modeling support what was observed, since the results were not nearly as clear in this model as shown for in vitro.

We thank the reviewer for these comments. We changed the color scheme in (new) Figure 4 (including panel AIII) to represent ECM and cell density more clearly. In the heading of Fig. 4C, we now state ‘Model prediction for percentage of interface migration’. However, we think that the color scheme on the right side of panel C helps to appreciate the percentage of interface migration/ total migration. As example, with MMPs inhibited, migration along the cleft becomes nearly 100% dominant at high, but not lower 3D collagen density. – Further, to improve accessibility to the readers, we separated the description of the mathematical model (in Methods)

© 2023. Published by The Company of Biologists under the terms of the Creative Commons Attribution License (https://creativecommons.org/licenses/by/4.0/).

from the results (in Results), and removed redundancies. Methods (page 14): ***'In silico model. To predict the effects of collagen density and collagenolysis on cell migration, an in silico cellular automaton was used (Deutsch and Dormann, 2018). The detailed mathematical procedures, simulation code and simulation videos were deposited at GitHub (https://github.com/TomPlt/Collagen-Interface-Model). Cell and ECM density of the experimental setup were represented by scalar fields defined on a 3D lattice with a lattice spacing of 10 µm. As initial condition, we set a plane of low ECM density representing the cleft that was surrounded by high ECM density, and a spherical volume representing the high cell-density spheroid that was placed in the middle of the lattice [Fig. 4A(I)]. Cell migration along the cleft, between the two interfaces, and invasion into the upper porous 3D matrix were modeled as a deterministic rule that incorporated proliferation, ECM proteolysis and interphase migration and depended on the local microenvironment defined by six neighboring lattice sites [Von Neumann neighborhood; Fig. 4A(II); shown are four neighbours].***
Proliferation was modeled as an increase in cell density according to the doubling time of MV3 cells. The assumption that cells were able to degrade surrounding ECM was implemented by a linear dependence of the ECM degradation rate on the cell density in the voxel and the neighboring voxels. For migration, two basic assumptions were made: (1) The surrounding ECM serves as a migration substrate and, when composed of linearly guiding low-density ECM regions, allows cells to adjust their movement direction; as example, increased cell flux during interphase migration is depicted in Fig. 4A(II).; (2) Cell migration is limited by available space and steric hindrance, which was implemented by a linearly decreasing relationship between outward migration rate and ECM density in neighboring voxels. This complex relationship between cell migration and the ECM neighborhood was implemented using a function with two regimes: (I) for low, increasing ECM densities in the near neighborhood, migration increases nearly linearly, with a maximum of this function being defined as the "critical ECM" density (Fig. 4A III, white zone) and and (II) for further increasing ECM densities in the neighborhood, migration speed decreases exponentially (Fig. 4A III, gray zone). By matching the experimentally observed relative and absolute cell numbers in the interfaces for high- and low-density collagen in a parameter scan, the model parameters were identified that best describe the experiments (Table 1; left): the high 3D collagen density was accompanied by a low density between the layers (ECM_{int} = 0.1), while the low-density assay resulted in a less well-defined boundary with a relatively higher cleft density (ECM_{int} = 0.26), whereas the critical ECM density was kept constant at ECM_{crit} = 0.1. Thus, consistent with their relative differences in collagen density in the cleft versus 3D collagen (Fig. S1, A-F), a well-defined and a less precise shallower interface was found for the high- and low- density condition, respectively. To predict the effect of MMP inhibition on cell migration into the different compartments of the ECM, the density of the ECM interface was fixed at the mean value (Table 1), and the 3D ECM density and degradation constants were systematically varied (Fig. 4C). Finally, the most likely combination of model parameters was identified for each experimental condition (Fig. 5J).' Results (page 7): ***In silico prediction of cell invasion in response to ECM density, topology, and proteolysis. To predict the consequences of ECM degradation for cell migration along the cleft, we applied an in silico cellular automaton model in which we simulated cell migration from a spheroid into the linear space between two 3D collagen meshworks (Fig. 4A(I)). Cell densities changed over time according to a deterministic interaction rule that incorporated proliferation, matrix degradation and ECM guidance (Figs. 4A II,III) and resulted in the characteristic tumor patterns observed in vitro (Fig. 4B; 4 movies at github link, see Methods). The model predicted that with increasing 3D ECM densities and decreasing degradation rates the percentage of cells migrating along the interface relative to all emigrating cells will increase further (Fig. 4C). When fitting the model to individual experiments, we found that cells had to degrade high-density collagen around 2.5 times faster as compared to low- density collagen, which was in good agreement with previous experimental findings (Infante et al., 2018). Taken together, the cellular automaton model allowed us to predict that without collagenolysis, migration along the interface between high-density collagen would continue and be most dominant.'.***

5. The weakness of in silico modeling is that the modeler can often adjust parameters to fit any results obtained from experiments. The great value of such computational modeling is the ability

© 2023. Published by The Company of Biologists under the terms of the Creative Commons Attribution License (https://creativecommons.org/licenses/by/4.0/).

to make testable predictions, ideally of findings that are not intuitively obvious. It would be most reassuring if the authors could use their model to make such a prediction and to test it.

We here ‘fitted’ the model with parameters on ECM density by the broad parameter scan. We then used this model to make predictions for experiments where proteolysis is inhibited. One of the predictions was that non-proteolytic cell migration along the interface of the high-density assay would still occur, and even remain dominant over non-proteolytic migration along the interface of the low-density assay (see new Fig. 5J), which in our opinion was not obvious. That our experiments confirmed these predictions shows the value of this mathematical approach.

6. The magnitudes of differences in interface migration with GM6001 treatment reported are not large, since Figure 4J has cut off 50% of the scale. They are nevertheless significant, but one is concerned that that result was due to the issue in point #3.

The reason why differences are relatively small results from the relatively low number of proteolytically invading cells into high- density 3D matrix. As a consequence, the ratio of interface versus all migrated cells in (new) Fig. 5J at baseline is 80%. Protease inhibition ablates 3D invasion completely, and residual MMP-independent migration, albeit reduced by 75%, occurred only into the cleft, which increased the ratio to 100% (Fig. 5J). We have changed in Fig. 5J the y-axis scale from 0 to 100%.

Additionally, the inhibition by GM6001 of interface cell number is quite strong, i.e., 80% in Supplementary Figure 4B. Is this really “MMP-independent migration”?

The reduction of migration along the cleft in the 6 mg/ml assay after MMP inhibition (Fig. S4B) is most likely caused by bridging collagen fibrils (see Fig. 1C) which, when not degraded, act as a moderate barrier. However, MMP-independent migration along the cleft in the low-density assay remained ~2 fold lower (see Fig. S4B). To clarify this point, we now state (page 7): ***‘Consistent with moderate hindrance imposed by the matrix evident with MMP inhibition...’ and ‘Last, at both high and low collagen densities, non-proteolytic cleft migration numbers and areas decreased by 50-75% when compared to proteolytic migration, and cells migrated similarly far (Figs. S4A,B; S5C,K; 5B-D). Interestingly, however, non-proteolytic migration into the ‘high-density’ cleft showed each a 1.7-fold increase of cell numbers and covered area, respectively, and thus a larger efficacy of residual jamming when compared to low-density collagen conditions (Fig. 5C,E; Fig. S4B,C).’***In context with the new data on migration across unconfined collagen, we now state (page 7/8***): ‘Consistent with moderate hindrance imposed by the matrix evident with MMP inhibition [7, 8] during cleft migration between high-density collagen lattices in the presence of GM6001, cell numbers, covered area, migration distance and invasion zone thickness decreased (Fig. S5 A-E,H and I-M,P), but not the cell density in the cleft itself (Fig. S5G,O). This jamming phenomenon, indicated by remaining multilayered migration, was again evident when compared to non-proteolytic migration of monolayered cells across the unconfined collagen surface (Fig. S5I-P).’ ... and .... ‘Interestingly, however, non-proteolytic migration into the ‘high-density’ cleft showed each a 1.7-fold increase of cell numbers and covered area, respectively, and thus a larger efficacy of residual jamming when compared to low-density collagen conditions (Fig. 5C,E; Fig. S4B,C). Thus, cell jamming depends on high spatial confinement but not MMP-mediated ECM degradation (Fig. S5H,Q).’***

Less important:

7. There seems to be increased density of collagen fibrils adjacent to the interface that is higher than the main collagen gel itself, e.g., in Figures 1B, 1C, Suppl. Figure 1A and 1E.

This is true. We now refer to the higher density by adding a small quantification on page 5 (referring to Fig. S1C; included in our answer to point 1 above).

Does this increased density affect cell behaviour, e.g., by forming a stiffer substrate?

Indeed, data from the literature may suggest that increased collagen density could result in increased stiffness and/or ligand density and thereby support migration. Increasing collagen density as applied in this manuscript and in previous own work associates with increasing

© 2023. Published by The Company of Biologists under the terms of the Creative Commons Attribution License (https://creativecommons.org/licenses/by/4.0/).

stiffness, ranging from 50 Pa (1.7 mg/mL) up to 600 Pa (8 mg/mL] [6, 7]. This stiffness increase favors tumor cell migration across a surface [9]. We now discuss this point as follows (page 9): ***'...In this previous work of ours, collagen concentration increases from 2 to 8 mg/ml induced an increase in stiffness from below 100 up to 600 Pa, a range that previously positively impacted cell migration rates (Ulrich et al, 2009) and may also promote cell migration across the lower interface boundary of the cleft.'***

8. This reviewer could not understand Figure 4 I, where the symbol legend for Control and GM-6001 and "Expe-riment" did not seem to make sense. What is the weak red-orange dashed line near the x-axis in the left graph mean? Might there have been a problem with producing the PDF for this figure?

Indeed, there was in part a technical problem when producing the pdf of (former) Figure 4, which we have revised for clarity.

9. It was not sufficiently clear what was meant by "matrix guidance" in this paper. At one point in the modeling, contact guidance is mentioned, but that is often a manner of migration following linear fibers or grooves. Elsewhere, the paper refers to a low density guiding interface, but is this just a narrow zone of low concentration collagen between two high concentration gels? Basically, what exactly is the mechanism of guidance, or is the term just used generally?

Thank you for raising this point. Indeed, we had used 'guidance' more as a general term, to describe migration along the interfaces. We have now replaced terms such as 'contact guidance' and 'matrix guidance' by 'interface guidance' or 'guided interface migration' to describe migration along the cleft.

Very minor:

19. It would have ideal for the authors to test more than one cell type, especially a "normal" cell to evaluate whether the findings are specific to cancer.

We agree. However, to set up this assay was not easy and cost a lot of time. We are happy to have received conclusive data on one cell line. Testing another cell line would significantly prolong the revision time.

20. On page 9, the text refers to "a number of around 2000" Is the word "cells" missing?

Yes, indeed. We included the word 'cells' (now on page 5).

It is hoped that the authors will be able to clarify these questions and resolve the concerns about this seemingly clever new assay.

*****

Reviewer 2 Advance Summary and Potential Significance to Field:
The authors developed a new method based on a 3D type I collagen assay containing a cleft-like interface between two lattices of collagen. They then studied the invasion of cancer spheroids into the gel. The interface has a higher porosity (authors claimed 2-10x increase which is quite heterogeneous). Based on their results and characterization, the authors claimed that this interface is "low-density guiding interface to disentagle the relative impact of ECM-derived barriers versus guidance cues". It appears that the guidance cues are of two kinds: a decrease of porosity at the interface but also a confinement between 2 layers of denser collagen gels. The main novelty of the paper relies on the development of a simple assay to guide cell invasion and migration.

Reviewer 2 Comments for the Author:
The authors developed a new method based on a 3D type I collagen assay containing a cleft-like interface between two lattices of collagen. They then studied the invasion of cancer spheroids into the gel. The interface has a higher porosity (authors claimed 2-10x increase which is quite heterogeneous). Based on their results and characterization, the authors claimed that this interface is "low-density guiding interface to disentagle the relative impact of ECM-derived

© 2023. Published by The Company of Biologists under the terms of the Creative Commons Attribution License (https://creativecommons.org/licenses/by/4.0/).

barriers versus guidance cues". It appears that the guidance cues are of two kinds: a decrease of porosity at the interface but also a confinement between 2 layers of denser collagen gels. The main novelty of the paper relies on the development of a simple assay to guide cell invasion and migration. The biological results are not new and supported by previous papers published by Friedl and Wolf's group and other studies.
Overall, I think that the paper may be of interest for the cell biology community and I could recommend its publication after some revisions.

Comments:
1/ SEM should be provided for both conditions (interface between dense 6mg/ml and less dense 2mg/ml collagen) and analyze pores sizes in these conditions. This is an important point to disentangle the impact of pore size and high/low-density ECM.

We indeed had undertaken various attempts to visualize low-density (2 mg/ml) interface collagen samples by SEM, but failed reproducibly, for technical reasons. The low-density gels were very fragile and collapsed during fixation, hence any space consideration was obsolete (see Figure R2, for the reviewers' discretion). Because sample shrinkage was a general issue, we abandoned the idea of obtaining quantitative data on pore dimensions from this approach We now infer the pore size indirectly, as follows (page 9): '*... **with an estimated 2-10 times increased pore size range when compared to the surrounding 3D collagen (Fig. 1C).***' (see also our answer 3 to reviewer 1).

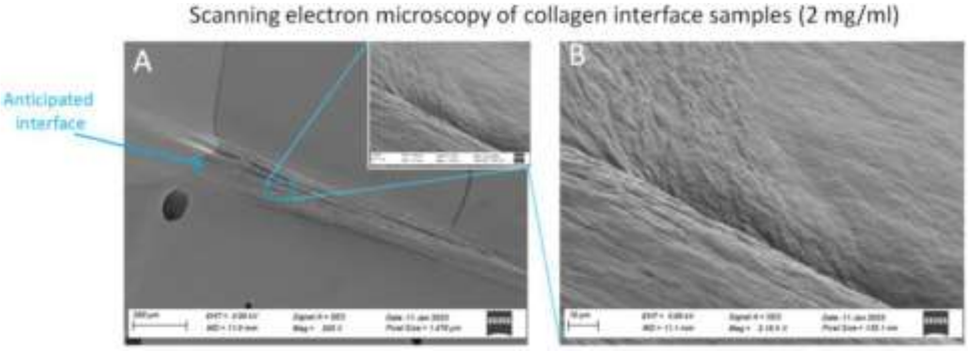

Figure R2.

2/ Concerning the inhibitor, I am wondering if the confinement of the cells could trigger the sensitivity to GM6001?

We have no indication to assume that the sensitivity to GM6001 would depend on collagen concentration or confinement. Because cells are mixed with collagen together with DMSO or GM6001, drugs are reaching the cells independent of collagen concentration, which differs from potential drug delivery issues in vivo caused by very dense ECM or high intra-tumor pressure.

What would be the effect of GM6001 on cell invasion in homogeneous large pore or small pores of 2mg/ml or 6mg/ml? Would migration patterns be the same (collective versus tracks)?

We addressed the dependence of mesenchymal cells in randomly organized fibrillar collagen in previous work ([6]; see accompanying Figure R3). When pores are large (i.e. in 2.5 mg collagen/ml gels), melanoma cells emigrate as strands or single cells from a spheroid, and this pattern is not affected by GM6001. In small pores (i.e. in 8 mg collagen/ml gels), however, proteolytic cells convert to exclusively collective migration, consistent with the 'jamming' phenotype observed in the preformed cleft (compare to Fig. 3E of this manuscript). In the presence of GM6001, cells stop translocating their main cell body by space restriction of the nucleus, consistent with the results in the current work in the randomly organized phase (6 mg/ml; see Fig. 5F).

© 2023. Published by The Company of Biologists under the terms of the Creative Commons Attribution License (https://creativecommons.org/licenses/by/4.0/).

Figure provided for reviewer has been removed. It showed Figure 3B from Haeger, A., et al., *Cell jamming: collective invasion of mesenchymal tumor cells imposed by tissue confinement*. Biochim Biophys Acta, 2014. **1840**(8): p. 2386-95. (doi: 10.1016/j.bbagen.2014.03.020)

3/The authors claimed that increased single cell invasion is observed at lower densities. It does not seem to be obvious from the current data. This point should be clarified.

We removed one of the arrows in Fig. 3E, lower right that did not clearly indicate a single cell. Into the sentence on page 6 we added the work 'occasionally', to tone down the appearance of single cells which was indeed not that obvious. The sentence reads now like this: '***In contrast, the 3D collagen compartment of the low-density assay was infiltrated by thin strands and, occasionally, single cells of both up to 1.5 x increased migration distance ... .***'

4/ For the modeling part, I think that the implementation of matrix degradation should be clarified. Furthermore, the authors claimed that they "identified the high 3D collegen density to be …" (P11 middle). I do not understand since it is implemented in the model and could not be an output. Further details are thus required.

Thank you for your comment. As outlined in reply #4 to reviewer 1, we re-wrote the modelling section for clarity. Indeed, ECM density was implemented in the model. To better represent ECM degradation, we increased the contrast in Fig 4A (II, ECM degradation). The unterlying mathematical terms were deposited at GitHub: https://github.com/TomPlt/Collagen-Interface-Model/blob/main/Math.%20Detail%20of%20the%20Sandwich%20Model.pdf We also identified the parameter combinations in silico that best matched the wet-lab data. We have slightly revised the Methods section, as follows (page 15***): ' To predict the effect of MMP inhibition on cell migration into the different compartments of the ECM, the density of the ECM interface was fixed at the mean value, and the 3D ECM density and degradation constants were systematically varied'***

5/ The authors claimed that they observed subcellular protrusions (p12 middle) of great length (Fig 4B, E). I had some difficulty to observe them. Could they provide better images?

We simplified this phrase by describing that cells moved 'similarly far'. On page 7, the new sentence reads like this: ***'Last, at both high and low collagen densities, non-proteolytic cleft migration numbers and areas decreased by 50-75% when compared to proteolytic migration, and cells migrated similarly far.'***

*****

Reviewer 3 Advance Summary and Potential Significance to Field:
In this manuscript, the authors present a model of spheroid migration at an interface of collagen. The interface presents the cells with a choice of dense or less dense(a "cleft") collagen in which to move. The authors then parse apart where cells move depending on the porosity and application of protease inhibitors. There has been significant work done in the area of proteolytic vs non-proteolytic migration in the field, by this group and others, and there has been work done previously investigating the behavior of cells at interfaces. While this manuscript presents a methodology to tune the interface, it is somewhat incremental and the results are fairly intuitive. So while interesting, the advance in the field is not as significant. The value of the model could be better demonstrated if some new biology is learned.

While PDMS-based interface models have been developed, we are not aware of an interface assay in which a cleft is formed by deformable 3D collagen of low or high density. This topology was "in-vivo"-inspired as it mimics tissue clefts observed in vivo, between collagen bundles, between collagen and myotubes, nerves fibers, and/or vessels. To clarify this point, we have revised the introduction, as follows (page 3): ***'To mimic an aspect of these versatile architectures present in interstitial tissues, reconstituted or cell-derived collagen-based models have been developed, ranging from 3D fibrillar networks of random orientation, varying porosity and fibril thickness to linearly aligned fibrillar structures, tunnel-like tracks in hydrogels', and under-collagen' assays'***. To further emphasise novelty, we now state (page 4): '***Whereas cell***

© 2023. Published by The Company of Biologists under the terms of the Creative Commons Attribution License (https://creativecommons.org/licenses/by/4.0/).

patterning into either tissue compartment, porous or lineary guiding, was studied before, how tumor cells choose ECM geometries for 3D invasion depending on the presence or absence of guidance cues and the ability to degrade ECM remains unclear. Thus, the assay developed here models a combination of geometrical structures which are amenable to MMP-dependent degradation, similar to connective tissues in vivo. - We respectfully disagree that the assay is not delivering novel biology. We here, for the first time, show that MMP inhibition can deviate invasion from dense to linear ECM compartments. In the revised manuscript we now strengthen the aspect that interference with MMPs delayed collective invasion but maintained cell jamming in the cleft (new Fig. S5F-H versus N-P), but not in high-density randomly organized collagen. In addition, the role of confinement in the cleft to induce multilayered cell jamming is now emphasized, in direct comparison to the monolayer migration emerging on the unconfined 2D collagen surface. Lastly, in silico modelling predicts these effects, as well as their adaptability across a broad parameter space of ECM density and ECM degradation. We have added these thoughts into different parts of the discussion section.

Reviewer 3 Comments for the Author:
The study is fairly straightforward. I have just minor comments:

Figure 1E: Authors state the cells spread to 6times the spheroid, however this value only has meaning when compared to the time allowed for spreading.

The 6-times spread occured over 48 hours. Because we have always used the same end-time, specifically 48 hours, we decided to leave our original labeling to keep things simple. However, we have clarified the 48-h-time in the text on page 5: ***'The interface assay enabled us to investigate the effect of ECM geometry on cell patterning over time, here over 48 hours (Fig. 1D, right; F).'***

Also, it is not clear what they mean by the spheroid not changing. Based on the images provided it is not possible to tell what the geometry or size of the spheroid core is compared to the cells migrating out from it. It appears to be expanding but it not clear how one defines the core.

Indeed, thank you for bringing this up. We have observed that the original spheroid does not seem to expand but somewhat flattens, i.e. from a sphere into an ellipsoid (see Figs. 1G, S2C). In some, but not all cases, an additional hollow space is formed around the original spheroid that separates the original core from the invading cells. Thus, we defined the rim of the spheroid core or, if present, the rim of the hollow space (see ellipse in cyan in revised Figure 1G) as reference to calculate cell migration. On page 5, we added this text accordingly: ***'Notably, the size of the spheroid remained largely constant over 24-48 hours, possibly due to effective cell emigration to a degree that an occasional hollow zone emerged (Figs. 1F,G, see ellipse in blue; S2A,C).'***

Figure 1F needs better labeling. Do the white areas indicate cuts?

Indeed. We have now labeled Figure 1F with the terms 'Cuts' and 'Scalpel', and added 'Cuts' to Figure S3A as well.
In general, the results should be reported as a function of time since all the differences in migration are measured for a given time period.
Please see our answer above.
A control should be run to ensure the inhibitors are working as desired (ie able to diffuse sufficiently to inhibit proteases as desired).

In Figure 5A, we show that GM6001 is working effectively (>90% inhibition of collagen degradation). As detailed in reply #2 to reviewer 2, cells, collagen monomers and GM6001 are mixed prior to collagen polymerization. Thus, the inhibitor does not need to diffuse and overcome potential spatial barriers to reach the cells. We also added the inhibitor to the supernatant at the same concentration as to the cell-collagen mix, so that its concentration is homogenous throughout the sample. On page 12, we had added this sentence in the previously submitted version: 'Where indicated, GM6001 or DMSO were supplemented to collagen lattices as well as supernatant.'

© 2023. Published by The Company of Biologists under the terms of the Creative Commons Attribution License (https://creativecommons.org/licenses/by/4.0/).

References

1. Beunk, L., et al., *Actomyosin contractility requirements and reciprocal cell-tissue mechanics for cancer cell invasion through collagen-based channels.* Eur Phys J E Soft Matter, 2022. **45**(5): p. 48.
2. Weigelin, B., Bakker, G.J., Friedl, P, *Intravital third harmonic generation microscopy of collective melanoma cell invasion -Principles of interface guidance and microvesicle dynamics.* IntraVital 2012. **1**(1): p. 1-12.
3. Ilina, O., et al., *Cell-cell adhesion and 3D matrix confinement determine jamming transitions in breast cancer invasion.* Nat Cell Biol, 2020. **22**(9): p. 1103-1115.
4. Zhao, Y., et al., *Automatic Segmentation of Cervical Cells Based on Star-Convex Polygons in Pap Smear Images.* Bioengineering (Basel), 2022. **10**(1).
5. Wolf, K., et al., *Multi-step pericellular proteolysis controls the transition from individual to collective cancer cell invasion.* Nat Cell Biol, 2007. **9**(8): p. 893-904.
6. Haeger, A., et al., *Cell jamming: collective invasion of mesenchymal tumor cells imposed by tissue confinement.* Biochim Biophys Acta, 2014. **1840**(8): p. 2386-95.
7. Wolf, K., et al., *Physical limits of cell migration: control by ECM space and nuclear deformation and tuning by proteolysis and traction force.* J Cell Biol, 2013. **201**(7): p. 1069-84.
8. Infante, E., et al., *LINC complex-Lis1 interplay controls MT1-MMP matrix digest-on-demand response for confined tumor cell migration.* Nat Commun, 2018. **9**(1): p. 2443.
9. Ulrich, T.A., E.M. de Juan Pardo, and S. Kumar, *The mechanical rigidity of the extracellular matrix regulates the structure, motility, and proliferation of glioma cells.* Cancer research, 2009. **69**(10): p. 4167-74.

Second decision letter

MS ID#: JOCES/2022/260207

MS TITLE: Cell jamming in a collagen-based interface assay: tuning by collagen density and proteolysis

AUTHORS: Lianne Beunk, Nan Wen, Sjoerd van Helvert, Bram Bekker, Lars Ran, Ross Kang, Tom Paulat, Simon Syga, Andreas Deutsch, Peter Friedl, and Katarina Wolf
ARTICLE TYPE: Tools and Resources

I am happy to tell you that your manuscript has been accepted for publication in Journal of Cell Science, pending standard ethics checks.

Reviewer 1

Advance summary and potential significance to field

Previous summary:
In this manuscript, the authors address an interesting question in cancer biology involving the behaviour of tumour cells in clefts or interfaces in vivo.
They develop a new assay in which a layer of collagen is polymerized first, then next a melanoma cell spheroid is positioned on this lower gel, and then a gel of the same concentration is poured and polymerized above it to produce to collagen layers with an interface of differing collagen. The authors find that the interface between these two gel layers has a significantly lower concentration of collagen with fewer fibrils. This interface or cleft is most apparent between 6 mg/ml high concentration collagen gels compared to 2 mg/ml collagen gels. The authors then quantify the expansion of cells from the spheroid, which is much greater along the interface than into the 3D gels facing each other. They provide evidence that MMP based proteolysis is most effective against 3D gel migration compared to interface migration, which they suggest may be relevant clinically in the sense that tumours invading in clefts or interfaces may be resistant to protease inhibitors. The experimental work is complemented by a computational model, which supports the findings.

© 2023. Published by The Company of Biologists under the terms of the Creative Commons Attribution License (https://creativecommons.org/licenses/by/4.0/).

The general question in cancer biology addressed by the authors involving the propensity of cells to invade along clefts or interstices and how this property can be elucidated is interesting and important. The assay described by the authors is potentially clever and interesting as a possible means of addressing these types of questions. The results presented seem to be of generally high quality and the illustrations are often excellent.

Comments for the author

The authors have responded directly, conscientiously, and satisfactoriy to my many suggestions and concerns. I am happy to recommend acceptance for publication in JCS.

© 2023. Published by The Company of Biologists under the terms of the Creative Commons Attribution License (https://creativecommons.org/licenses/by/4.0/).
